# PROTOCOL: Participation, inclusion, transparency and accountability (PITA) to improve public services in low‐ and middle‐income countries: a systematic review

**DOI:** 10.1002/CL2.205

**Published:** 2018-07-30

**Authors:** Hugh Waddington, Jennifer Stevenson, Ada Sonnenfeld, Marie Gaarder

## Background

### The problem of unaccountable government systems and poor service delivery

The sustainability of global development investments depends on strong institutions, citizen engagement, accountable governments, and equitable economic growth (World Bank, 2017). Goal number 16 of the Sustainable Development Goals explicitly recognises the importance of the development of effective, accountable and transparent institutions at all levels, and of ensuring responsive, inclusive, participatory and representative decision‐making at all levels (UNDP, 2016). In the Paris Declaration on Aid Effectiveness, donor and partner countries committed to improving their mutual accountability and transparency in the use of development resources, with partner countries further committing to systematically involve diverse stakeholders in national development priority setting processes (OECD 2005). Many development challenges, such as poor service delivery, corruption and slow growth, persist because of the political context around them; they are as much about power dynamics as they are technical challenges.

Improving the governance of public institutions and service delivery has long been a central tenet of strategies for achieving or supporting development; World Bank *World Development Reports* since the late 1990s have included elements of improving governance as central to their theories of change (Grindle, 2004). In the decades since, mainstream approaches to realising good governance have shifted in focus, away from privatisation of service delivery and towards a focus on increasing the engagement of constituents, particularly vulnerable groups, with public institutions and service providers in such ways to increase the effectiveness, appropriateness, and quality of service delivery. The 2004 *World Development Report* (WDR) highlighted the insight that public spending on service delivery in developing countries often primarily reached the better‐off minority of citizens; for example, in India, curative health subsidies were primarily going to the richest 20 per cent of the population, who received three times the subsidies of the poorest 20 per cent (World Bank, 2004). This insight remains pertinent. For example, a recent evaluation of an e‐governance intervention in India that aimed to improve transparency in a fiscal transfer system for a social benefits programme suggested that while the intervention was successful at reducing leakages, the savings did not translate into improved outcomes for beneficiaries (Banerjee et al., 2017). One of the authors later posited that this may have been because the intervention did not empower the ultimate beneficiaries to ensure that financial gains from reduced corruption were converted into increased outcomes for the poor (Page and Pande, 2018).

There are many definitions of governance. For the purposes of this review, we use the recent definition employed by the World Bank, where governance is defined as “the process through which state and non‐state actors interact to design and implement policies within a given set of formal and informal rules that shape and are shaped by power” (World Bank, 2017). Where characteristics of good governance are weak or absent from public processes and service delivery, the effectiveness and sustainability of development interventions is likely to suffer (World Bank, 2016). Barriers to access to public services for vulnerable groups exacerbate inequality, with potential long‐term repercussions for a society's development (Easterly, 2007). Fraud and corruption are pervasive across low‐ and middle‐income countries, and the negative consequences on quality of life and core development outcomes are well documented (Svensson, 2005; Molina et al., 2016). Where state and public actors cannot be effectively held accountable, a culture of impunity develops that normalises fraud and rent‐seeking practices. The World Bank's *World Development Report 2017* highlighted key repercussions of power asymmetries, including: exclusion of large portions of society from services, institutions or resources, which is correlated with violent conflict: elite and/or interest‐group capture of policies in order to serve interests, resulting in poor targeting and ineffective or inappropriate policies, which can lead to poor or stagnant growth, condemning economies to an under‐developed state; and clientelism, which often leads to rent‐seeking and poor service delivery, which have long‐term repercussions on societies’ growth (World Bank, 2017).

Despite the decades of acknowledgement of the importance of good governance, progress has been slow; the Worldwide Governance Indicators show limited to none or even negative progress on key governance indicators amongst aggregates of low and lower‐middle income countries from 2006 to 2016 (World Bank, 2018). The repercussions of continued governance failures are high, and well documented; for example, in Nigeria, unabated corruption led to the squandering of billions of dollars by the National Petroleum Company, jeopardizing the country's long‐term growth potential and financial stability (World Bank, 2017).

Approaches to improve governance have generally either focused on mechanisms to strengthen the effectiveness and institutionalisation of public institutions, or on external pressures to improve service delivery despite weak institutions. While each approach has yielded valuable insights, translating insights from theory into practice has been challenging. There is some evidence that at times, failures could be due to an over‐emphasis of the demand side of governance by service users, citizens and civil society, which ignores the constraints faced on the supply side by politicians, bureaucrats and service providers (Brinkerhoff and Wetterberg, 2015), or of the power of information (Wibbels and Keohane, 2018). More recently, insights are emerging into the value of system‐based approaches that look at both the supply and demand sides of governance as actors in a single system, drawing on power analyses and social network theories (Mcloughlin and Batley, 2012; Fox, 2014; Halloran, 2015; Wibbels and Keohane, 2018).

USAID's Democracy, Human Rights and Governance (DRC) Center identified *participation, accountability, transparency and inclusion* (PITA) as critical principles that could be incorporated into interventions within and across sectors to improve development outcomes, and in line with the Doing Development Differently global initiative (USAID, 2016). We define participation as efforts to involve citizens in the design, monitoring and delivery of policy and programmes upstream (Quick and Feldman, 2011). Transparency is a “characteristic of governments, companies, organisations and individuals of being open in the clear disclosure of information rules, plans, processes and actions” (Transparency International, 2009: 44). Accountability is the concept that individuals, agencies and organisations are held responsible for executing their powers according to a certain standard downstream (McGee and Gaventa, 2011). Finally, inclusion means a particular focus on marginalised and vulnerable citizens in policy and programming upstream or downstream (Quick and Feldman, ibid).

### Interventions promoting participation, inclusion, transparency and accountability

#### Mapping the universe of PITA interventions

A recent evidence gap map (EGM) on interventions for state‐society relations highlighted a number of interventions to improve governance (Phillips et al., 2017). These were broadly grouped into interventions for inclusive political processes and leadership (e.g. community‐driven development, electoral monitoring, and quotas for women and minority representation in political institutions), and interventions for responsive and accountable institutions and service delivery (e.g. audits, land reform and public servant performance incentives).

Drawing on Phillips et al. (2017), and also insights from the literature, we theorise good governance can come about through sustained improvements across three domains: within the political system; within the management and administration of public sector offices and institutions; and in the ways in which public officials and service providers engage with service users (external engagement). In this framing, good governance interventions attempt to influence the social contract that mediates the relationships between government and citizens, regarding who has access to what power and in return for what accountability for service provision, through three accountability domains:

- *Influencing how the broader political system functions:* The broader political system dictates access to and contestability of the policy arena (World Bank, 2017). It further comprises the checks and balances, or “sideways accountability” between institutions. Increasingly, good governance interventions seek to influence *how* this system functions, rather than the specific form it takes (World Bank, 2017).
- *Influencing how a specific public service or institution's system functions internally:* Many good governance interventions aim to improve service delivery through the institutionalisation of public services and institutions. These interventions foster “internal accountability” of institutions, and include, but are not limited to, strengthening human resources management, systems of upwards accountability of staff and management or between different levels of government, and supply chains for infrastructure, goods, and financial flows (Finan, Olken and Pande, 2015).
- *Influencing how a specific public service or institution engages externally with constituents:* These interventions aim to mediate the ways that citizens engage with government and public service providers outside of the “long route” of electoral processes (World Bank, 2004). They work to improve service delivery through “external accountability”, by increasing the engagement between service providers and service users to improve the responsiveness and effectiveness of public services.

**Figure 1:**
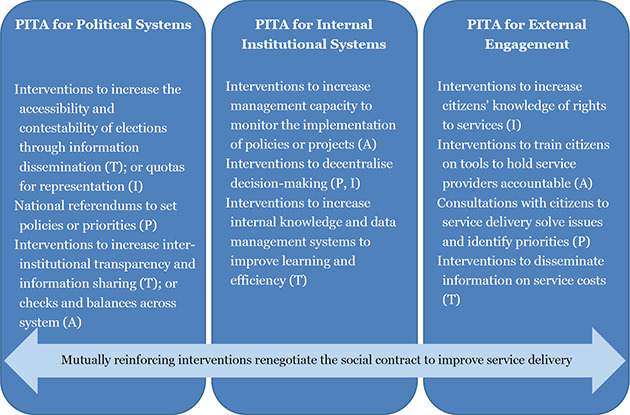
PITA interventions throughout the three domains of good governance Source: Authors

Many good governance interventions are designed to improve service delivery for citizens. This is often done through interventions that embody one or multiple PITA characteristics, which seek to address power dynamics between the state, civil society and citizens to make service delivery more effective and equitable (USAID, 2016). PITA characteristics influence the functioning of the social contract and its systems throughout each of the three accountability domains, and thus, good governance interventions may target one or more of these (Figure 1). For example, within the political system domain, the PITA characteristics have a direct impact on who has access to the electoral systems and who can contest the policy arena. Elected officials must exercise some basic level of downwards accountability towards the constituents who elected them (or, in non‐democratic states, who grant them legitimacy), and sideways accountability to their fellow statesmen through the checks and balances built into the system. PITA interventions targeting this domain tend to focus on *creating a fair system*. Within the internal system domain, PITA interventions tend to focus on *creating an efficient system*, such as through improving the upwards accountability of officials and service providers to management, or through improving the relevance of service provision at local levels through decentralisation. Finally, in the external engagement domain, the PITA characteristics of a service or institution mediate the means through which it engages with citizens, civil society, and business/interest groups. These PITA interventions aim to address a more diverse set of system attributes, primarily the *relevance, effectiveness and inclusivity of the service delivery system*, and are further differentiated from those in the previous domains through their reliance on soft power.

The effectiveness of interventions that target the PITA characteristics within one domain will be mediated by the context of the other domains as well, the power relations and constraints, and also by other interventions aiming to improve good governance and service delivery, particularly those that target service delivery supply chains. There is increasing scholarship that suggests that while interventions improving the PITA characteristics of public services and institutions, particularly in the external engagement domain, may be *necessary* for achieving sustainable improvements in service delivery and a stable social contract, they may not be *sufficient* (e‐Pact Consortium, 2016). On the other hand, while interventions that target strengthening PITA characteristics within internal institutional systems may be sufficient for improving governance within the system, the impact of those governance improvements may not reach the ultimate beneficiaries (citizens/service users) without the incorporation of interventions strengthening the system's external PITA characteristics (Page and Pande, 2018).

While recognising the interactions of PITA interventions across each domain and with complementary good governance and service delivery initiatives, it has been pointed out that to attempt to cover the entirety of good governance interventions in a single review would be “exceedingly ambitious” (Sáez, 2013). Thus, this review will analyse the value‐add of interventions in the third domain, external PITA interventions targeting public service and institution engagement with citizens.

#### The focus of the review on PITA for external engagement with service users

External PITA interventions can be implemented as stand‐alone interventions or as part of a larger programme working to strengthen governance and service delivery. They may be implemented either on the supply or demand side of service delivery, or may target both simultaneously, such as a public audit process that trains community members on tools to hold public officials accountable, and works with public officials to increase their understanding of the importance of downwards accountability and transparency. As in that example, the interventions may strengthen one or multiple PITA characteristics of the ways public services and institutions engage with their constituents.

**Figure 2:**
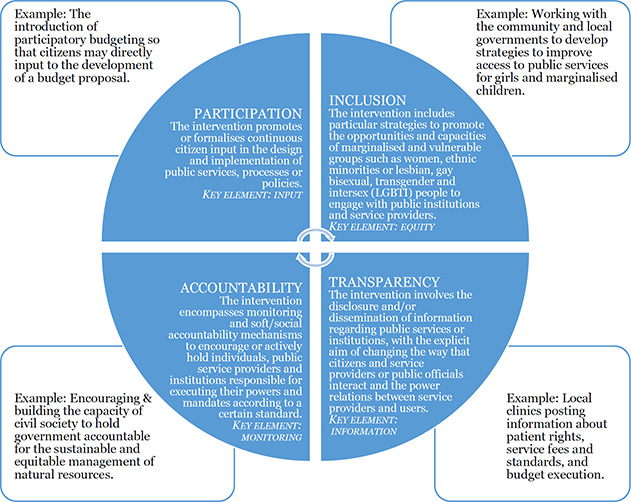
Operationalising external PITA into intervention categories Source: Authors

For the purposes of this review, the definitions of PITA have been further operationalised as follows (Figure 2):

- *Participation:* The intervention promotes or formalises continuous citizen input in the design and implementation of public services, processes or policies.
  ∘ Participation interventions create specific opportunities or processes for citizens to provide meaningful input into public policy or strategy design and planning. An example of a participation intervention is the introduction of participatory budgeting so that citizens may directly contribute to the development of a budget proposal (Touchton & Wampler, 2014). A community‐level example could be the creation and capacity building of a representative community‐based natural resource management committee that is mandated to develop and monitor locally agreed standards and regulations for the use of common property.
- *Transparency:* The intervention involves the disclosure and/or dissemination of information (rules, plans, processes, prices and actions) regarding public services or institutions, with the explicit aim of changing the way that citizens and service providers or public officials interact and the power relations between service providers and users.
  ∘ An example of a transparency intervention is local clinics posting information about patient rights, service fees and standards, and budget execution (USAID, 2016), which restricts the scope for service providers to charge bribes..
- *Accountability:* The intervention encompasses monitoring and soft/social accountability mechanisms to encourage or actively hold individuals, public service providers and institutions responsible for executing their powers and mandates according to a certain standard.
  ∘ Accountability interventions create opportunities or processes for constituents to hold government and public service providers accountable. An example is a project to encourage and build the capacity of civil society to hold government accountable for the sustainable and equitable management of natural resources (USAID, 2016), or a citizen report card intervention, in which a community group is taught the quality standards to which they are entitled and how to monitor the quality and performance of service delivery, and then to work with the service providers to address any identified issues through a mutually agreed action plan.
- *Inclusion:* The intervention includes particular strategies to promote the opportunities and capacities of marginalised and vulnerable groups such as women, ethnic minorities or lesbian, gay bisexual, transgender and intersex (LGBTI) people to engage with the management of public institutions and service providers. Hence, we define inclusion specifically as a component of an intervention that targets a change in participation, transparency or accountability.
  ∘ An example of an intervention to promote inclusion is ensuring that a certain proportion of places in a community governance group are reserved for women (Humphreys et al., 2012).

The intervention categories are described in more detail in (Table 3) in the Methodology section below. While most interventions contribute primarily to a single PITA mechanism as described above, there is often significant interplay between the PITA characteristics to which an intervention contributes. Though efforts have been made to make the definitions mutually exclusive, a single intervention may contribute to strengthening multiple PITA characteristics. For example, a public audit may improve the community's understanding of services the government can and cannot provide (contributing to transparency), while further allowing them an opportunity to pressure and apply informal sanctions to public officials should the audit uncover discrepancies in spending (contributing to accountability). More generally, interplay at outcome level often exists within transparency and accountability dimensions. For example, interventions to improve access to information about users’ rights to services (transparency) may aim, at an intermediate outcome level, to improve the way a service is governed (accountability).

As noted above, according to the definition used in this review, eligible interventions that are designed to improve the access of a marginalised group of citizens (inclusion) to a decision‐making process aim, at an intermediate outcome level, to improve the group's input into the process by providing increased opportunities for consultation (participation), or other aspects of monitoring service delivery (transparency and accountability). Indeed, the assignment of other intervention sub‐types will vary depending on their particular iteration within a project. For example, officials may mandate a health care committee to increase access to health services for marginalised community members through community campaigns (contributing to inclusion) and/or to undertake health service monitoring (contributing to accountability). Thus, the ultimate assignment of a study looking at a health care committee intervention will depend on the structure and mandate of the committees within that project.

### How PITA interventions might work

**Figure 3:**
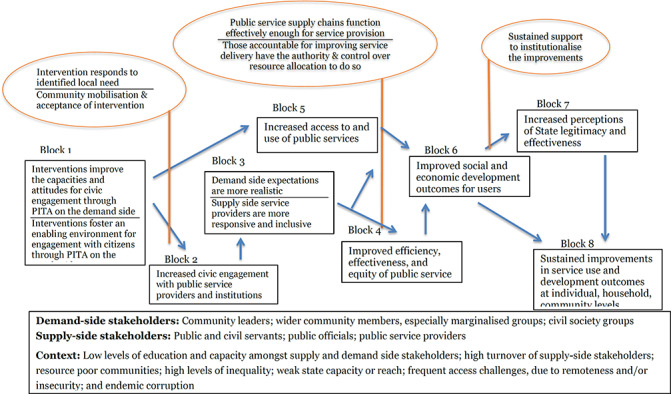
Intervention theory of change

We present a model showing an indicative, stylised theory of change for how the intervention may work (Figure 3). This preliminary theory of change developed for this systematic review draws on insights from the literature and programmatic best practices. In particular, the framework builds on the 2004 WDR (World Bank, 2004) theory of change, which articulated the importance of pro‐poor governance practices that actively engage end users for effective outcomes, and Rahman and Robinson (2006) who articulated the importance of local ownership and long‐term support. The assumptions and moderating factors draw on insights from Fox (2014), Page and Pande (2018), and the 2017 WDR (World Bank, 2017), among others.

This review does not take a “rights‐based approach” that views improvements in PITA characteristics as the end objective. While recognising the value of PITA characteristics in and of themselves, the focus of this review is on the value‐add they bring to improving development outcomes through improved service delivery. It is unlikely that any single theory of change would be able to capture all the ways different PITA interventions affect good governance outcomes and service delivery. Thus, this review, at this protocol stage, attempts instead to hypothesise the main causal pathways through which PITA interventions targeting public services’ and institutions’ external engagement with citizens lead to improved development outcomes. Some interventions may contribute to all the pathways; others may only contribute to one. Similarly, as the focus is on the external engagement processes, while some interventions may have effects on other domains of the social contract such as electoral systems, they are not captured in this theory of change. The theory of change will be built out and developed in more detail through the evidence synthesis.

We note here a useful distinction between the demand and supply side of governance. Implementers may target stakeholders on the demand‐side of governance, such as through efforts to improve the capacity of civil society to monitor government service delivery, or the supply‐side, such as by training public officials on pro‐poor development planning. Other interventions may be geared to affecting both demand‐ and supply‐sides, such as a participatory budgeting process in which government officials are trained on the value of participatory budgeting, while community members are trained and supported to participate in the process.

**Demand‐side stakeholders:** Community leaders; wider community members, especially marginalised groups; civil society groups

**Supply‐side stakeholders:** Public and civil servants; public officials; public service providers

**Context:** Low levels of education and capacity amongst supply and demand side stakeholders; high turnover of supply‐side stakeholders; resource poor communities; high levels of inequality; weak state capacity or reach; frequent access challenges, due to remoteness and/or insecurity; and endemic corruption

The indicative theory of change presents the hypothesised causal chain for PITA interventions, from changed opportunities and capacities, followed by behavioural changes on both the supply and demand side of governance, ultimately leading to improved service delivery performance and enhanced quality of life outcomes for citizens.

Beginning with the intervention on the left hand side, the figure follows a primary causal chain, with immediate, intermediate and endpoint outcomes indicated in boxes, and key assumptions in bubbles. The theory of change starts with critical assumptions of the design, inception and implementation phases: first, that the intervention designed is relevant and addresses an identified local need; second, during inception that wider community acceptance for the intervention has been sought and received from key social, religious and political leaders; and finally, that community mobilisation activities are undertaken during implementation. Similar to how the quality of PITA characteristics in the public planning and service delivery spheres contributes to strengthening the corresponding development outcomes, the strength and quality of the PITA characteristics of the intervention itself are suggested to contribute to its efficacy.

The exact form of the intervention (Block 1) will vary widely, yet the majority aim to create an enabling environment for increased and mutually empowering interactions between service providers and citizens through changes to their knowledge, attitude and practices (KAPs). On the demand side, this may include efforts to improve citizens’ knowledge of the services to which they are entitled; their capacity to demand those services through key tools; and/or their sense of self‐efficacy and empowerment to do so effectively. An intervention focused on a technical skill such as participatory budgeting may start with capacity building on budgeting processes and the role that citizens can play; interventions that aim to increase inclusion of marginalised groups often start with community campaigns to raise awareness amongst the target households.

On the supply side, either in addition to demand‐side efforts or independently, interventions aim to strengthen openness from and active engagement with supply‐side stakeholders in efforts to improve service delivery. These may target the actors implementing or managing the service in question, but also other key stakeholders in the community and throughout the system. Seeking and attaining community acceptance prior to implementation is a widely‐applied best practice for ensuring that development projects do no harm and that they will have sufficient buy‐in from the community to be successful. There is some evidence that suggests that this may be particularly critical for PITA interventions. Securing buy‐in from stakeholders at the point of intervention, upstream and downstream along the service delivery / good governance supply chain may create an enabling environment for PITA interventions to successfully navigate the social network and power differences within which the intervention is implemented (Mcloughlin and Batley, 2012).

Different tools implemented as elements of PITA interventions may require different conditions to be effective; the synthesis process will aim to identify and these out. The context will also influence what works, how; for example, if a political player can increase his or her own personal power through framing an improvement in service delivery as a personal “win,” then she or he may be more motivated to work for its improvement (e‐Pact, 2016).

The first immediate outcome (Block 2) posits that through engaging in the PITA interventions, citizens will increase their engagement with State and public service officials. This is often an explicit aim of PITA interventions, as it is a critical precursor to the higher‐level outcomes. Through the increased engagement, the next level of change (Block 3) posits that citizens develop a better understanding of processes, services, and the constraints faced by service providers, while simultaneously, service providers gain a deeper understanding of the needs of their constituents and appreciation for the engagement process.

In subtle ways, these changes reflect renegotiations of power relations between the State, civil society and citizens, mitigating the power imbalances. This happens as the PITA interventions shift the dynamics of power by drawing on collective and representative voice.

- Participation interventions address power relations by building in meaningful opportunities for citizens to provide input over the direction of policies that affect them and the supply of services they rely on. For example, participatory development planning interventions enable community groups to develop their own plans and priorities, which are then consolidated into a coherent local development plan, which then feeds up into higher‐level plans. This ultra‐decentralised decision‐making ensures that the priorities of target communities are at the forefront of any final government development plan.
- Inclusion interventions address power relations by bringing marginalised voices to the table. While there is some debate as to whether or not this leads to meaningful opportunities to provide input, there are other very worthwhile effects; for example, the presence of a person from a marginalised group on a body that handles conflict resolution can significantly increase the access to that service for other members of the marginalised group.
- Transparency interventions address power relations by limiting the government and public service providers’ capacities to use their positions for personal gain, and addressing the power difference caused by knowledge gaps. By improving the dissemination and publication of public sector processes, it becomes harder for officials to take advantage of vulnerable participants; if someone knows how much a service is supposed to cost, and the official knows the person knows, then the official is more likely to provide the service at the correct cost.
- Finally, accountability interventions address power relations by increasing the risk and severity of informal social sanctions against poorly performing bureaucrats and service providers.

Power relations are dynamic; they can change quickly, both for the better and worse, and gains are not necessarily secure. A key assumption here is that supply‐side actors are fully engaged throughout the process; otherwise, the attempts to increase soft power by citizens may be seen as confrontational rather than collaborative, which could de‐incentivise service providers from the process to avoid being seen to give up any of their power (World Bank 2004). Where PITA processes are seen as collaborative, they can be mutually empowering, creating changes in the interactions between state and society that simultaneously give citizens greater input into the provision of the services they rely on, and strengthen the standing of the service providers in the community (Fox, 2014).

Where interventions are unsuccessful at building coalitions to facilitate an enabling environment for change, they may not be successful at changing power relations, as actors may adapt to new systems (Halloran, 2015). For example, though advancements in the field of information and communications technology (ICT) offer exciting possibilities for strengthening external PITA characteristics, a change in technology that is not complemented by supporting interventions that create an enabling environment may fall flat (Hogge, 2010).

As the power relations are shifting and engagement is increasing, a core intermediate outcome of the PITA interventions will emerge (Block 4): public service delivery will improve in efficiency, effectiveness, and equity. Once public officials and service providers are taking into account the input of community members, the selection and targeting of services will improve. This will improve the effectiveness and appropriateness of public service delivery. Inclusion interventions improve the equality of service provision, as they increase access to services and processes for the most vulnerable community members. Transparency initiatives increase the efficiency of public service delivery, as they streamline costs and processing times, and make it harder for politicians and officials to demand inflated payments for services. Finally, accountability initiatives can have direct benefits to the performance of public service delivery, as citizen feedback mechanisms such as Public Audits end with joint workshops between the service provider, citizen representatives, and other key stakeholders to come up with an actionable plan to which all parties can be held to account for how they will address the major issues identified and improve service delivery.

The key assumption here is that institutions have the capacity to respond to priorities requested and issues raised by constituents. This is a critical assumption, because in its absence, the interventions risk doing harm by having a negative consequence on perceptions of State effectiveness resulting from raised and then unmet expectations. For example, the 2017 WDR highlights the risk that investments in service provider capacities may not be enough to improve service delivery, if power relations within the institution are not addressed (World Bank, 2017). Further, depending on the structure of the intervention, improvements may be related to a one‐off change in the situation that is not sustained; many PITA interventions are designed as experiments, whose study design may capture short‐term gains that revert back to the baseline conditions with time. Fung et al. suggest that transparency interventions contribute to improvements only when the information provided becomes embedded in the decision‐making process (2005).

In some cases, PITA interventions, particularly those that focus on improving access to services for marginalised groups (I), may not lead to the active, empowered engagement between citizens and service providers that leads to mitigated power differences and improved services. However, they could still lead to increased use of public services, particularly amongst vulnerable populations (Block 5). This comes about as a direct result of Inclusion and Transparency (information dissemination) interventions, but also through the other PITA interventions; as communities are mobilised to engage with their local government and services, they become more invested in the services that they are attempting to improve. And thus, they are more likely to take advantage of those services, as they understand the importance of ensuring high quality service provision for themselves and their families. However, increased access for marginalised groups is not a given outcome of PITA interventions; there likely needs to be concerted, targeted efforts to reach and engage these groups in order for the impacts to reach them (E‐Pact Consortium, 2016). Similarly, interventions targeting services where changes are relatively immediate and visible may be more likely to encourage buy‐in and support from supply‐side actors (ibid.).

The joint effects of changes from Blocks 4 and 5 lead to improved development outcomes for citizens (Block 6). These will vary by sector targeted, and by project duration. It is increasingly thought that PITA interventions, through the immediate outcomes increasing engagement with government and public officials and mitigating the power differences, can have a positive impact on perceptions of state effectiveness when services and development outcomes improve as a result (World Bank, 2017) (Block 7). As citizens engage with public processes and services, they learn more about the constraints under which these institutions operate. As they see increased responsiveness of public officials, and subsequent real improvements in the quality of services they receive, their perceptions of State effectiveness and legitimacy will increase. This is particularly critical in fragile and post‐conflict Sates, where the State may still be vying with other actors for legitimacy over governing and control.

The social contract, wherein the state and citizens negotiate the granting of power and legitimacy in return for service provision, is a complex system that is being constantly renegotiated as actors at various levels within and outside of the government interact and negotiate for their own individual power and accountability, within frameworks of collective power and accountability. Thus, it is both the context within which good governance interventions are implemented, and also at the heart of the systems and services that said interventions aim to improve. There is some evidence that the relationship between politics and service delivery is two‐way: the political context influences service delivery, but service delivery also influences politics, particularly state legitimacy (Mcloughlin and Batley, 2012). Thus, all good governance interventions in some way influence the social contract between the state, its institutions and service providers, and the citizenry; on the other hand, good governance failures often stem from fragmented social contracts (World Bank, 2017). This perspective also draws on the ideas of a social accountability ecosystem developed by Halloran (2015).

In the majority of PITA programmes, the PITA characteristics interventions are add‐ons to core interventions and outcomes in a public service sector, such as health or agriculture. In the long run, all three intermediate outcomes contribute to sustaining social and economic outcomes of communities (Block 8). The key assumption here is that sustained support is provided to the institutions or service providers charged with maintaining the implementation of the PITA intervention, such that it becomes institutionalised. As noted above, power differences are dynamic and constantly evolving. Thus, a short‐term project may well change outcomes in the short‐term, but without proper support those gains may easily be lost. Thus, sustainability mechanisms must be built into the PITA programme design, to support communities and service providers on the road to sustainable participatory, inclusive, transparent and accountable public service delivery and public management.

The context in which this theory of change, or elements of the same, are implemented has strong ramifications on the ways in which the interventions must be designed, implemented and supported in order to ensure success. Common key characteristics are noted in the box below the theory of change, alongside the key stakeholders from both the supply and demand side of governance. Governance programmes are generally implemented in resource‐poor contexts, where there are entrenched problems around low levels of education and capacity, high turnover amongst public officials, and endemic corruption. Target communities are frequently difficult to access, either due to remoteness and extreme weather, or to conflict and insecurity. It is precisely because of these challenges that governance interventions are so strongly needed in such areas, but they must be taken into account during the design phase to ensure risks are appropriately mitigated. These factors breed vicious cycles of weak public service supply, which leads to weak demand, which in turn facilitates weaker public financial management, and so on. In an ideal world, the PITA interventions would create a virtuous circle of active community engagement in their government and service provision.

Interventions tailored to the specific context in which they are implemented, that target both the demand and supply sides of good governance, are more likely to be successful, particularly when the interventions are supplemented by complementary ones that target the technical side of service delivery and/or service delivery supply chains. For example, in the Philippines, a project focusing on improving access and quality of maternal and child health and family planning included social accountability mechanisms in the form of Quality Assurance Partnership Committees, which Brinkerhoff and Wetterberg (2015) argue led to more effective service delivery that improved the client‐focus of providers and increased service use.

Additional moderating factors that will have a strong influence on project results include top‐down political will; key to ensuring that local government officials and service providers have the capacity to implement the changes they agree to with their constituents is having the support of the higher levels of government, which can ensure that funds are appropriately allocated. Political will further influences the sustainability of the results, and the possibility of a change in administration poses a risk to programmes that may be cut due to high association with the outgoing regime.

It is important at this point to also highlight two broad issues which determine the effectiveness of programmes, relating to intervention design and implementation fidelity. There are two main reasons why we might not expect to see the intended impacts of a programme implemented in the ‘real world’ (Bamberger et al., 2010). The first is that the programme design is inappropriate – that is the underlying mechanisms that drive change are not appropriate for the context in which the programme is based, or for particular groups of participants in that context (Pawson, 2006). According to van der Knaap et al. (2007: 3), “mechanisms are the engines behind behavior, which are often not immediately recognizable… They [include] people's efforts to give way to group pressure (groupthink), people's efforts to be status‐congruent with others or to avoid or reduce cognitive dissonances, or people's desire to be an early adopter of an innovation. [T]he action of mechanisms to some extent depends on the context in which they are used… Behavioral change is achieved through this context”.

An example would be a community driven development programme that is supposed to rely on community participation to foster social cohesion, but is unable to support the appropriate level of participation, and therefore cohesion, because people are not comfortable speaking in public meetings due to elite capture (Snilstveit, 2012; White et al., 2018). Similarly, interventions to decentralise decision making in schools are less likely to be effective in low income, low education contexts where communities have low status relative to school staff (Carr‐Hill et al., 2016). Another example would be a women's empowerment programme which is ineffective in reaching a particular sub‐group of participants (e.g. women from Muslim households) because it does not take into consideration the need to involve community leaders in design of the programme targeting strategy.

Such “failure mechanisms” will vary based on intervention design and targets; for example, in some cultures, traditional community leaders may be critical stakeholders to engage in interventions seeking to change the equity of or access to services, despite the disconnect between their de facto and de jure power – but only depending on the service targeted. Baldwin and Raffler (2016) argued that traditional leaders are often highly socially accountable for public services such as conflict resolution or natural resource management, but less so for services such as education or health care. In that case, an intervention targeting equitable access to and use of public land may fail if it does not engage traditional leaders, but a similar intervention simply targeting equitable access to and use of health services may still be successful. Failures may also come in the form of unintended consequences; for example, Chong et al. (2014) found that increasing the dissemination of corruption information to voters in Mexico decreased support not only for exposed corrupt politicians, but also for all political parties, and led to a decrease in voter turnout.

The second reason is due to implementation failures for a programme that otherwise (in theory) would be effective in the implementation context. Examples would be technical and logistical problems relating to project delivery (e.g. inadequate training and support to practitioners); weaknesses in implementer systems (e.g. human resource, financial or monitoring); or due to external factors (e.g. conflict, natural disasters).

#### Why it is important to do the review

The 2017 *World Development Report* (World Bank, 2017) posits that rather than asking which policies to implement, the global development community needs to ask what enables policies to achieve sustainable outcomes, the answer to which being better governance. This report is timely in a context in which donors are actually moving away from funding governance projects. Data from the OECD Creditor Reporting System of Official Development Assistance (ODA) shows declining funding in the government and civil society sector, from US$ 14.5 billion in 2009 to around US$ 12 billion in 2015, a decrease of almost 20 per cent. During the same time period, overall ODA increased from US$ 103 billion to US$ 118 billion (OECD, 2017), so the share of aid to governance and civil society also fell from around 14 per cent to 10 per cent.

Governance programmes are implemented in complex socio‐political contexts, and involve many challenges in realising, demonstrating, and attributing improvements towards key outcomes. USAID notes that the lack of consistent definition of governance and poor understanding and weak documentation of evidence of governance‐related interventions contribute to a reticence to invest in such programmes (2017). This could explain why donors are shifting their attention towards other sectors; over the same time period (2009 to 2015), funding for economic infrastructure and services increased by US$ 7.5 billion, while funding for health programmes increased by US$ 700 million (OECD, 2017).

In addition, prominent single study evidence has questioned the viability of bottom‐up, community‐based approaches, as compared to top‐down government accountability (Olken, 2007). However, it is not clear whether the findings from single studies are transferable to other contexts. This points to the need to strengthen the synthesis and dissemination of the evidence base, and to encourage decision makers to draw on systematic evidence collected from multiple contexts.

This systematic review will broadly look at interventions that promote more effective and responsive public services and institutions, defined under Sustainable Development Goal number 16 as institutions that “deliver equitable public services and inclusive development at the central and local levels, with a particular focus on restoring core government functions in the aftermath of crisis and attention to local governance and local development” (UNDP, 2016).

The evidence gap map on interventions to improve state‐society relations concluded that there are few systematic reviews on governance topics, and many of those that are available are out‐dated or of low quality in terms of the methodology they employ (Phillips et al., 2017). Phillips et al. identify several high‐quality existing systematic reviews that cover evidence that is relevant to the substantive scope of this review. Many of these have focused on governance interventions in the education sectors. Relevant non‐systematic evidence syntheses include Olken and Pande (2013), Azulai et al. (2014), Dal Bo and Finan (2016) and the Metaketa project (EGAP 2018). More recently, a study for USAID combining literature review and key informant interviews (Brinkerhoff et al., 2017) identified studies examining accountability in low‐ and middle‐income country health services and synthesised the programme mechanisms.

A recent systematic review of education interventions (Snilstveit et al., 2015) examines the effects of several governance related interventions in the education sector, including community‐based monitoring, school‐based management, providing information on returns to schooling and monitoring of teacher attendance. This is a mixed‐methods review which draws on impact evaluations and other relevant literature to examine the full causal chain. Similarly, Carr‐Hill et al. (2015) produced a review on interventions to shift decision‐making to the school level and the impacts this has on educational outcomes. The authors included both impact studies and other empirical research to study barriers to, and enablers of, effective school‐based management. They include a total of 26 impact evaluations and nine qualitative studies, finding decentralisation is effective in middle‐income contexts but not generally effective in low‐income contexts where parents are not able to hold school decision‐makers accountable.

Molina et al. (2016) undertook a systematic review focusing on community monitoring studies in the health, education and infrastructure sectors on a range of outcomes including corruption, access to health and education, test scores and mortality rates. The authors incorporated 15 impact evaluations and related quantitative and qualitative literature in an attempt to synthesise evidence around the circumstances in which, and why, programmes lead to better or worse outcomes.

There are also two systematic reviews of community‐driven development (CDD) (King et al., 2010; White et al., 2018). In CDD interventions, community groups are empowered to manage small‐scale infrastructure projects and basic service provision directly, from prioritisation and project selection through to implementation, through capacity building and financial support. CDD interventions typically contain a participation and inclusion intervention plus a block grant that is used to build community infrastructure. The study by White et al. (2018) combines a theory‐based synthesis using qualitative data synthesis with statistical meta‐analysis to assess the effects of CDD on social capital and cohesion on the one hand, and its effects on infrastructure creation, access and quality of life outcomes on the other. Most evaluations of CDD assess the impact of CDD as a whole and not the differential effect of the PITA component from the infrastructure block grant.

Phillips et al. (2017) identify several other systematic reviews that cover relevant literature. These include Lynch et al. (2013), who produced a systematic review of the effect of interventions that improve community accountability on service delivery and corruption; Hanna et al. (2011), who look at anti‐corruption interventions;^1^ and Guerrero et al. (2012), who focus on teacher monitoring and incentives to improve accountability.

The existing reviews overlap to some extent with the substantive focus of the proposed review, but several do not follow the Campbell guidelines for the production of systematic review, and the searches for many are now out of date. Further, none of the existing systematic reviews focus explicitly on studies that make a distinction between an intervention or policy with PITA characteristics and a comparison group that experiences a policy without PITA characteristics or weaker characteristics. Most importantly, no extant review has focused on governance interventions and articulated the PITA mechanisms through which programmes operate.

As policy makers and implementers work to ensure the sustainability of their investments and interventions, institutionalising good governance practices will become increasingly important. This systematic review will assess the effectiveness of interventions that target participation, inclusion, transparency and accountability in the design and delivery of public services and institutions on development outcomes. Analysis of causal pathways and mechanisms will shed light on the contexts in which these interventions can be successful and corresponding enabling factors. The review responds to a demonstrated need for evidence of what is generalisable, what is context specific, in what ways, and for whom in governance programming.

## Objectives

The main objective of this review is to identify, appraise and synthesise evidence that answers the question: to what extent are programmes targeting effective and responsive public services and institutions that incorporate PITA characteristics into their design effective in achieving their objectives, as compared to otherwise similar programmes that do not? We will compare the effectiveness of different types of programmes that incorporate PITA characteristics, both by intervention type, for example information dissemination intervention or community based monitoring, and by which PITA mechanism(s) the intervention incorporates. We will aim to answer the following specific review questions:

1. What are the effects of interventions that aim to strengthen the PITA characteristics of public services or institutions on social and economic wellbeing for participants? (*Review Question 1*).
2. What are the effects of interventions that aim to strengthen the PITA characteristics on participatory, inclusive, transparent or accountable processes? (*Review Question 2*).
3. To what extent do effects vary by population group and location? (*Review Question 3*).
4. What factors relating to programme design, implementation, context, and mechanism are associated with better or worse outcomes along the causal chain? (*Review Question 4*).
5. What evidence is available on programme costs and incremental cost effectiveness in included studies of effects? (*Review Question 5*).

## Methodology

The review will follow Campbell and Cochrane Collaborations guidance for systematic reviews (The Steering Group of the Campbell Collaboration, 2016; Kugley et al. 2017; Higgins & Green, 2011; Shadish & Myers, 2004). The review will also draw on guidance for theory‐based impact evaluation (White, 2009), theory‐based systematic reviews (Waddington et al., 2012; Snilstveit, 2012) and realist synthesis (Pawson, 2006; van der Knaap et al., 2008). To address questions 1,2 and 3, we will draw on counterfactual evidence provided in quantitative causal studies (impact evaluations) and use analysis of effect size data (statistical meta‐analysis) to explore the central tendency and heterogeneity for outcomes measured along the causal chain. The review will draw on multiple types of evidence, including programme and project documents to provide information about context and mechanism characteristics, and implementation processes (question 4). The review will also draw on cost data to the extent these are available (question 5). Cost effectiveness is a key question for decision makers, and one that is rarely incorporated into systematic reviews.^2^

### Criteria for including and excluding studies

The study inclusion and exclusion criteria are summarised in Table 1.

**Table 1:** Summary of criteria for inclusion and exclusion of studies

**Criteria**	**Inclusion definition**
**Population**	**Programme participants in LMICs are included. Programme participants in high‐income countries are excluded.**
**Interventions**	**Interventions with PITA components that target the means and mechanisms through which public institutions and services engage with constituents (service users) are included. Interventions that bundle PITA components alongside other programme components such as block grants (e.g. community‐driven development), or that aim to strengthen internal or sideways PITA, or those in the education sector are excluded.**
**Comparisons**	**Populations that receive ‘business as usual’ service access, or an intervention with a different type or degree of PITA are included. Comparators that receive no intervention at all are excluded.**
**Outcomes**	**Intermediate and endpoint, intended or unintended outcomes at participant and project level are included. Outcomes relating to political processes (e.g. voting) are excluded. Immediate outcomes relating to citizen engagement (e.g. participation in meetings) or bureaucrat responses (e.g. public spending) are eligible for the review provided that outcomes relating to access to services (e.g. facilities construction) or intermediate outcomes (e.g. service use) or final outcomes (e.g. health or nutrition) are also reported.**
**Study design**	**Counterfactual studies (research questions 1‐4), including relevant programme and project documents providing information on design and implementation (research question 4) and cost evidence provided in counterfactual studies (research question 5) are included.**

#### Types of study designs

To answer research questions 1, 2 and 3 we will include counterfactual studies that use an experimental or quasi‐experimental design and/or analysis method to measure the net change in outcomes that are attributed to an intervention or policy. We will include randomised and non‐randomised studies that are able to take into account confounding and selection bias (Reeves et al., 2017; Waddington et al., 2017). Specifically, we will include the following study types:

- Randomised controlled trials (RCTs), with assignment at individual, household, community or other cluster level, and quasi‐RCTs using prospective methods of assignment such as alternation.
- Non‐randomised studies with selection on unobservables:
  ∘ Regression discontinuity designs, where assignment is done on a threshold measured at pre‐test, and the study uses prospective or retrospective approaches of analysis to control for unobservable confounding.
  ∘ Studies using design or methods to control for unobservable confounding, such as natural experiments with clearly defined intervention and comparison groups, which exploitnatural randomness in implementation assignment by decision makers (e.g. public lottery) or random errors in implementation, and instrumental variables estimation.
- Non‐randomised studies with pre‐intervention and post‐intervention outcomes data in intervention and comparisons groups, where data are individual level panel or pseudo‐panels (repeated cross‐sections), which use the following methods to control for confounding:
  ∘ Studies controlling for time‐invariant unobservable confounding, including difference‐in‐differences, or fixed‐ or random‐effects models with an interaction term between time and intervention for pre‐intervention and post‐intervention observations;
  ∘ Studies assessing changes in trends in outcomes over a series of time points (interrupted time series, ITS), with or without contemporaneous comparison (controlled ITS), with sufficient observations to establish a trend and control for effects on outcomes due to factors other than the intervention (e.g. seasonality).
- Non‐randomised studies with control for observable confounding, including non‐parametric approaches (e.g. statistical matching, covariate matching, coarsened‐exact matching, propensity score matching) and parametric approaches (e.g. propensity‐weighted multiple regression analysis).

Analysis under research question 4 aims to address programme design, implementation, context and mechanism in greater detail. We will draw on a wider range of evidence on programme design and implementation. We will therefore incorporate descriptive information about a programme evaluated in one of the included counterfactual studies, as well as from programme and project design and implementation documents.

Analysis under research question 4 aims to integrate evidence on design, implementation, contexts and mechanisms with evidence of effects. Information on underlying context and behavioural mechanisms may draw on information contained anywhere in included study reports, whereas evidence on outcomes will draw on effects data from quantitative counterfactual estimation.

Analysis under research question 5 aims to address unit cost, cost‐efficiency, cost‐effectiveness or benefit‐cost evidence on interventions in particular contexts. We will incorporate economic evaluations of included programmes drawing on standard approaches to synthesis of economic appraisal evidence (Shemilt et al., 2011). Evidence included will incorporate unit and total costs to implementers and participants (and non‐participants, as relevant).

#### Types of comparisons

Eligible comparators for research questions 1‐3 include groups that receive normal service delivery (‘business as usual’) without improved PITA characteristics, or groups that receive an intervention testing the inclusion of different PITA design characteristics or weaker or less intensive implementation of PITA design characteristics. Table 2 presents examples of studies employing different types of comparison groups to illustrate these distinctions. While we have made distinctions here between comparison groups receiving an intervention with no explicit PITA characteristics versus a group receiving an intervention with weaker PITA characteristics, it may be difficult to determine the distinction in practice due to problems in implementation or reporting.

**Table 2:** examples of eligible comparison groups

**Type of comparison group**	**Example study**
**A group experiencing business as usual service delivery (no PITA intervention)**	**Pandey et al. (2008) ‐ India: Comparison between a group of villages that received public meetings to disseminate information on entitled health services, entitled education services, and village governance requirements – compared to control villages that received equivalent public service provision entitlements but no information.**
**A group with an intervention with different PITA characteristics**	**Nyqvist Bjorkmann et al. (2014) ‐ Uganda: Comparison between a group receiving report cards and one receiving report cards plus information about performance.**
**A group with an intervention with weaker PITA characteristics**	**Finkel (2012) ‐ Democratic Republic of Congo: Comparison between a group receiving workshops organised by community organisations to stimulate discussion and learning about on‐going decentralisation – compared to a group experiencing decentralisation but did not get offered a workshop.**

#### Types of participants

We will include any participants from low‐and middle‐income countries (L&MICs), including participants from the general population and those from specific population sub‐groups. We will collect data on differential effects and experiences for sub‐populations available and code information according to the PROGRESS‐plus criteria, where progress stands for place of residence, race/ethnicity, occupation, gender, religion, education, socioeconomic status, and social capital, and ‘plus’ represents additional categories such as age, disability, and sexual orientation (O'Neil et al. 2014).

#### Types of interventions

To be included in the review, the intervention must meet one or more of the following characteristics to improve the effectiveness and responsiveness of institutions’ engagement with constituents:

- *Participation:* The intervention promotes or formalises continuous citizen input in the design and implementation of public services, processes or policies.
- *Inclusion:* The intervention promotes engagement with the whole community in public service governance, particularly that of marginalised and vulnerable groups such as women, ethnic minorities or lesbian, gay bisexual, transgender and intersex (LGBTI) people.
- *Transparency:* The intervention involves the disclosure and/or dissemination of information (rules, plans, processes, prices and actions) regarding the governance of public services or institutions, with the aim of changing power relations between service providers and users.
- *Accountability:* The intervention encompasses monitoring to encourage or actively hold individuals, public service providers and institutions responsible for executing their powers and mandates according to a certain standard.

Specifically, we will include PITA interventions that aim to mediate the external engagement by public institutions and services with the citizens they serve. We define PITA interventions as either stand‐alone interventions or interventions that form part of a larger programme, that inherently or by definition seek to improve the PITA‐characteristics of engagement between public services and institutions and citizens. They may be implemented either on the supply or demand side of service delivery, or may target both simultaneously, for example through the introduction of public‐service audits that work with both the community and civil servants. Interventions targeting education service provision are excluded, as we believe high‐quality systematic reviews exist on PITA interventions within this sector (Snilstveit et al., 2015; Carr‐Hill et al., 2016), even if they may be in need of search updates.

**Table 3:** Examples of external PITA interventions

**Intervention group**	**PITA characteristic(s)**	**Target stakeholders**	**Examples** ^*^
**Sector‐specific community‐based committees**	**P, I, T, or A**	**Demand‐side**	Healthcare Committees that improve knowledge of health services and encourage their use (P) Water User Associations that improve the equitable distribution of water resources (I) Community Development Councils that institutionalise participatory development planning processes (T, A)
**Political inclusion**	**P or I**	**Supply‐side**	Strengthening the motivation and capacity of public officials to enforce rights to consultation for communities affected by proposed extractive industry projects (P) Quotas for women's representation in government bodies or public processes (I)
**Public service assessments**	**A**	**Demand‐side**	Citizen Report Cards, in which a community group gathers data on service delivery quality from service users, analyses and presents findings to the service provider
**Community‐based audits**	**T or A**	**Demand‐side & Supply‐side**	Public Audits, in which a government line department presents their budget and achievements to their constituents and engages them in a process of review to identify opportunities and create a plan for improvement
**Community‐based monitoring**	**A**	**Demand‐side**	Participatory Monitoring Committees, in which a diverse group of community members is trained on the services and quality to which they are entitled, and mechanisms for monitoring the delivery
**Simplified service delivery**	**I**	**Supply‐side**	Single‐Stop Service Centres, in which officials from diverse government line departments and services are brought to a single location (often in a remote area) to lower barriers to access and improve consistency in service delivery
**Standardised service delivery**	**T**	**Supply‐side**	Standard‐Service Delivery Boards that publicise standardised costs and processing times for key services
**Information dissemination**	**I or T**	**Demand‐side & Supply‐side**	Community Campaigns to increase knowledge of available services or public processes and promote participation
**Good governance training**	**P, I, T, or A**	**Supply‐side**	Exposure visits for district officials to learn about pro‐poor governance (I) Training for provincial line department staff on the importance and value of downwards accountability (A)

* *Note: examples are not exhaustive, but intervention groups are. A longer list of examples was used to develop the search strategy*.

#### Types of outcome measures

We will include studies that report outcomes measuring improvement in access to services, service behaviours, and social and economic quality of life improvements for the proposed intervention. Our inclusion criteria for outcomes are broad in order to be able to provide a full picture of the effects of the included interventions along the causal chain, described in Table 4.

**Table 4:** Examples of outcomes along the causal chain

**Causal chain area**	Service user engagement	Service provider response	Service access	Service use	Attitudes to services	Wellbeing outcomes
**Example**	Participation in meetings	Public spending	Facilities construction (quantity and quality)	Use of services	User satisfaction	Socio economic outcomes, sustainability

*Primary outcomes:* intermediate outcomes that measure service access and use (block 5 in the theory of change), and endpoint outcomes that measure social or economic wellbeing for individuals (block 6), user satisfaction (block 7) and sustainability (block 8) in the relevant sector, are eligible. Examples of well bing outcomes are: morbidity or mortality; income, wealth or poverty status; nutritional status or food security; resilience to shocks; crime rates.

*Secondary outcomes:* ‘immediate outcomes’ measuring citizen engagement with public institutions and services, such as participation in decision‐making, inclusion, transparency and accountability, and responsiveness of public services and public service delivery agents, such as public spending, leakages and corruption.

#### Duration of follow‐up

We will include any follow‐up duration, coding multiple outcomes where studies report multiple follow‐ups.

#### Types of settings

Interventions may be implemented in any low‐ or middle‐income country, as defined by the World Bank at the time the intervention was implemented.

#### Other

We will include both completed and ongoing studies, for example protocols of ongoing studies that appear to meet all other inclusion criteria or studies listed in registries of ongoing impact evaluations. This is a rapidly expanding field and listing ongoing studies will give an overview of where current knowledge gaps might be filled in the future.

We will include studies published in any language, although search terms will be in English only. We will include studies published in 2000 or after, following Phillips et al. (2017).

### Description of methods used in primary research

We will follow the inclusion and exclusion criteria outlined in the previous section to include studies in the systematic review. We anticipate that we will identify a mixture of experimental and quasi‐experimental studies. Examples of studies included and excluded are given in Appendix 2, and include the following:

- Finkel (2012), “*The VOICE Impact Evaluation in the Democratic Republic of the Congo Final Report*”. This study uses an experimental design and is an example of a PITA intervention using information dissemination around on‐going decentralisation in the Democratic Republic of Congo
- Touchton & Wampler (2014), “*Improving social well‐being through new democratic institutions”*. This study uses a quasi‐experimental design (propensity score matching) and is an example of a PITA intervention to introduce participatory budgeting in Brazil.

In addition, interventions conducted in schools and education facilities, or education outcomes measured in otherwise eligible interventions, will be excluded. Two examples are:

- Aturupane et al. (2014), “*An Assessment of the Impacts of Sri Lanka's Programme for School Improvement and School Report Card Programme on Students’ Academic Progress*”.
- Nguyen et al. (2013), “*Information, Role Models and Perceived Returns to Education: Experimental Evidence from Madagascar*”.

### Search strategy: Studies to address review question 1

We developed the systematic search strategy in consultation with an information specialist (John Eyers) to cover comprehensively the published and unpublished literature, following systematic search guidelines in Kugley et al. (2017)). We also drew upon, and expanded, the search terms used in the evidence map by Philips et al. (2017) and harvested terms from the papers included in that map that were eligible for inclusion in our review. To reduce the potential for publication bias, the search will include both academic databases as well specialist organisational websites, websites of bilateral and multilateral agencies and repositories of impact evaluations in international development. The full list of databases is presented below. The substantive scope of this review is cross‐sectoral and therefore in addition to general sources of social science research, we will search several sector specific databases, for example databases of health, governance and public management. We will search for studies published in 2000 or after up until 2018.

Search terms for the academic databases can be found in Appendix 1. Separate search strings were developed for the two academic health databases to capitalise on MeSH terms, to remove non‐health related terms and add some specific health‐related intervention terms (Medline and Global Health). The search strings combine specific intervention terms, study design terms and terms for low‐and middle‐income countries.

A simplified series of search strings was developed for searching the grey literature, wherein the search engines are not as sophisticated as the academic databases and cannot handle the same detailed strategy. Due to the broad scope of the review, and in order to ensure the grey literature search was exhaustive, a series of PITA search strings were developed. These focused on PITA terms such as participatory or participation. An intervention‐based strategy, more similar to the academic database strategy, was piloted, but discarded due to the number of individual searches per site that were required for an exhaustive search, rendering it inefficient. Population and study type terms were not included, because the advanced search options within the grey literature search engines were not sophisticated enough to allow for an “or” limiter for each LMIC and methodology. The broad study type term “impact evaluation” was added alongside each search to improve the relevance of results.

#### Electronic searches

We will search the following academic databases:

- CAB Global Health (Ovid): http://www.ovid.com/site/catalog/databases/30.jsp
- Econlit (Ovid): http://www.ovid.com/site/catalog/databases/52.jsp
- Medline (Ovid): http://www.ovid.com/site/catalog/databases/901.jsp
- Scopus: https://www.scopus.com/
- Social Sciences Citation Index (SSCI) (via Web of Science): https://webofknowledge.com/.

We will search the following specialist organisational databases:

- CARE International: http://www.careevaluations.org/
- Catholic Relief Services: https://www.crs.org/our‐work‐overseas/research‐publications
- Centre for Public Impact: https://www.centreforpublicimpact.org/observatory/
- Chemonics International: https://www.chemonics.com/technical‐areas/democracy‐and‐governance/
- EGAP (Evidence in Governance and Politics): http://www.egap.org/
- International Growth Centre (IGC) at LSE: https://www.theigc.org/publications/
- International Rescue Committee (IRC): https://www.rescue.org/reports‐and‐resources
- Mercy Corps: https://www.mercycorps.org/research
- Oxfam International: https://policy‐practice.oxfam.org.uk/publications
- RTI International: https://www.rti.org/publications
- Samuel Hall (evaluations): http://samuelhall.org/category/publications/
- Transparency International (TI): https://www.transparency.org/
- U4 Anti‐Corruption Resource Centre: http://www.u4.no/publications/.

Bilateral and multilateral agencies and general repositories of impact evaluations in international development to be searched include:

- 3ie Repository of Impact Evaluations http://www.3ieimpact.org/en/evidence/impact‐evaluations/
- 3ie RIDIE (Registry for International Development Impact Evaluations): http://ridie.3ieimpact.org/
- African Development Bank (AfDB):https://www.afdb.org/en/documents/publications/
- Asian Development Bank (ADB):https://www.adb.org/publications
- BREAD:http://ibread.org/bread/papers
- Center for Effective Global Action (CEGA):http://cega.berkeley.edu/evidence/
- Design, Monitoring and Evaluation for Peace: www.dmeforpeace.org/learn/resources/
- DFID Research for Development (R4D): http://r4d.dfid.gov.uk/
- GEF (Global Environmental Facility) evaluation database: http://www.gefieo.org/evaluations/all?f[0]=field_ieo_grouping%3A312
- Global Facility for Disaster Reduction and Recovery: https://www.gfdrr.org/en/publication
- Innovations for Poverty Action (IPA): http://www.poverty‐action.org/projectevaluations
- Inter‐American Development Bank Publications: https://publications.iadb.org/facet‐view?locale‐attribute=en&field=type_view
- J‐Poverty Action Lab (J‐PAL): https://www.povertyactionlab.org/evaluations
- Global Facility for Disaster Reduction and Recovery: https://www.gfdrr.org/en/publications
- Locus (International Development Coalition):https://locus.ngo/resources
- Prevention Web (UNIDSR): https://www.preventionweb.net/english/professional/
- RePEc (via EBSCO Discovery): https://www.ebscohost.com/discovery
- World Bank E‐Library (via EBSCO Discovery): https://www.ebscohost.com/discovery
- United Nations Evaluation Group: http://www.uneval.org/evaluation/reports
- USAID Development Clearing House: https://dec.usaid.gov/dec/home/Default.aspx.

#### Other searches

Our primary source of existing studies and reviews will be the evidence gap map of state‐society relations that preceded this systematic review (Phillips et al., 2017). In addition, we will also screen the bibliography of existing systematic reviews and literature reviews for eligible studies, including Molina et al., (2016), Lynch et al. (2013) and Hanna et al. (2011). We will also screen the reference lists of included studies and undertake forward citation‐tracking for those studies using Google Scholar. Forward and backward citation tracking has already been undertaken for the studies from Philips et al. (2017) which provided the original framework for this review. Therefore, we will only undertake forward citation tracking for included studies going forward from the date on which that exercise was previously done.

We will contact key experts and organisations through our review advisory group (details provided in acknowledgements) to identify additional studies.

### Targeted search for studies to address review question 4

In order to answer question 4 relating to programme design, implementation, mechanisms and context, we will attempt to identify programme and project documents associated with the programmes in the impact studies identified in the first stage of the search. We will do this by undertaking a targeted search for programme names and authors using Google Scholar. Evidence on context and mechanisms will be collected from any studies eligible for research questions 1‐4. Programme mechanisms may be suggested by study authors or identified by the review team. In addition, we may impute contextual information not provided in included studies using international data, for example the World Development Indicators (World Bank) or the “Polity IV” governance index (Marshall et al. 2011).

### Studies to address review question 5

We will incorporate economic evaluations and cost data that are provided in the included impact studies.

### Screening

All search results will be imported into EPPI‐Reviewer 4 and duplicates removed. All studies will be double screened in EPPI reviewer 4 against the review inclusion criteria by two independent reviewers using information available in the title and abstract. Any disagreements will be resolved by a third reviewer. If a title and abstract does not present enough information to definitively include or exclude a study, it will be included for full‐text screening.

At the title and abstract stage, we intend to use innovative text mining technologies to reduce the initial screening workload (O'Mara‐Eves et al., 2015). We will use two functions in EPPI Reviewer 4 to do this: the priority‐screening function and inclusion/ exclusion classifier (Thomas et al., 2011; O'Mara‐Eves et al., ibid). The priority screening function can be used at the title and abstract screening stage to prioritise the items most likely to be ‘includes’ based on previously included documents. This will involve double screening a random test set of 100 citations to train the priority screening function, which will learn to identify relevant records based on key‐words in the title and abstract of the included and excluded studies. All team members will be involved at this stage of screening. The function continues to learn as screening progresses. Using priority screening in this way allows for the identification of includable records at an earlier stage in the review process so that work can begin earlier on full‐text screening and data extraction. We will also use the priority screening function to develop a classifier that will classify studies into groups based on their probability of inclusion in the review. We will conduct piloting and verification, but we anticipate excluding studies with a low probability of inclusion (<10% probability of inclusion) automatically from the review. We would screen a random sample of the automatically excluded studies as a check on accuracy of the function.

Studies included for full‐text screening will be double screened by two independent reviewers. Disagreements on inclusion or exclusion will be resolved by discussion and the input of a third reviewer if necessary.

Screening of studies intended to address research question 4 will take place in a second stage of screening in EPPI reviewer. Studies will be assessed for relevance, that is, whether they cover one of the programmes covered by the included impact evaluations, and whether they meet the minimum quality requirements, described in the section below on assessment of quality in qualitative studies and process evaluations.

### Criteria for determination of independent findings

We will report data according to the intervention that the evidence was based on. We will attempt to avoid double‐counting of evidence and synthesis of dependent findings from multiple studies in any single analysis by linking papers prior to analysis, and conducting sensitivity analysis as necessary. Where information is collected on the same programme for different outcomes at the same or different periods of time, we will extract information on the full range of outcomes over time. Where multiple outcomes are reported from different specifications, we will select the specification according to likely lowest risk of bias in attributing impact, for example the most appropriately specified outcomes equation. Where studies reported multiple outcome sub‐groups for the same outcome construct (e.g. studies reporting simple, intermediate and complex knowledge), we may calculate a “synthetic effect size”. Where studies report multiple outcomes or evidence according to sub‐groups of participants, we will report data on relevant sub‐groups separately. Further information on criteria for determining independent effect sizes is presented below.

### Data extraction and coding procedures

We will extract the following descriptive, methodological, qualitative and quantitative data from each included study using a standardised data extraction form (provisional form provided in appendix 2):

- Descriptive data including authors, publication date and status as well as other information to characterise the study including country, type of intervention and outcome, population, context, type of intervention.
- Methodological information on study design, analysis method, type of comparison (if relevant) and external validity.
- Quantitative data for outcome measures, including outcome descriptive information, sample size in each intervention group, outcomes means and standard deviations, test statistics (e.g. t‐test, F‐test, p‐values, 95% confidence intervals), cost data, and so on.
- Information on intervention design, including how the intervention incorporates participation, inclusion, transparency and accountability characteristics, participant adherence, contextual factors and programme mechanisms.

We will extract quantitative data for outcomes analysis using Excel. We will extract descriptive, methodological and qualitative data using KoBo Toolbox. Descriptive and qualitative data will be single coded by one reviewer and checked by a second reviewer.

### Critical appraisal

For critical appraisals, we will report the full results for each study included in the final review.

#### Assessment of risk of bias in experimental and quasi‐experimental studies (Review Questions 1‐3)

We will assess the risk of bias in the included quantitative counterfactual studies (impact evaluations) drawing on the signalling questions in the 3ie risk of bias tool which covers both internal validity and statistical conclusion validity of experimental and quasi‐experimental designs (Hombrados and Waddington, 2012) and the bias domains and extensions to Cochrane's ROBINS‐I tool (Sterne, Higgins et al., 2016). Two reviewers will undertake the risk of bias assessment independently. If there are disagreements, they will be resolved by discussion and the involvement of a third reviewer as necessary. The provisional risk of bias tool can be found in appendix 3. We will do the risk of bias at the paper level, noting any potential differences in methods and risk of bias by different outcome.

We will assess risk of bias based on the following criteria, coding each paper as ‘Yes’, ‘Probably Yes’, ‘Probably No’, ‘No’ and ‘No Information’ according to how they address each domain:

- Factors relating to baseline confounding and biases arising from differential selection into and out of the study (attrition);
- Factors relating to biases due to deviations from intended interventions (e.g. performance bias and survey effects) and motivation bias (Hawthorne effects);
- Factors relating to biases in outcomes data collection (e.g. social desirability or courtesy bias, recall bias);
- Factors relating to biases in reporting of analysis.

We will report the results of the assessment for each of the assessed criteria for each study.

In addition, we will attempt to explore if there are systematic differences in outcomes between primary studies with different risk of bias. If meta‐analysis is feasible, we will conduct sensitivity analysis to assess the robustness of the results to the risk of bias in included studies.

#### Critical appraisal of project design and implementation (Review Question 4) and cost evidence (Review Question 5)

It is not necessary to critically appraise the information that we will extract on programme design, implementation and context that comes from the project documents as this information is descriptive. To assess the quality of the cost evidence, specifically cost effectiveness studies, cost‐benefit or cost‐efficiency studies we will draw on the approach to the appraisal of cost evidence taken in Doocy and Tappis (2016). They adapt two guides to the use and appraisal of cost evidence; the German Federal Ministry for Economic Cooperation and Development's Tools and Methods for Evaluating the Efficiency of Development Interventions (BMZ, 2011) and the Campbell Collaboration Economic Methods Policy Brief (Shemilt, 2008). Following this approach, we will rank cost evidence into three levels by their analytic power: high level (studies of cost‐effectiveness analysis, cost‐benefit analysis, cost‐utility analysis); medium level (cost analyses, cost‐comparison studies, cost‐outcome descriptions); and low level (descriptive / raw cost data associated with an intervention evaluated in one of the impact evaluations only). We will then use the checklist laid out in Shemilt et al. (2008) to appraise the methodology of level 2 and level 1 studies, which includes assessment of both cost and effectiveness components of the analysis.

### Effect size calculation

An effect size expresses the magnitude or strength of the relationship of interest (Borenstein et al., 2009; Valentine et al., 2015). To address questions 1, 2 and 3, we will extract data from each individual study to calculate standardised effect sizes for cross‐study comparison where possible. For continuous outcomes comparing group means in a treatment and control group, we will calculate the standardised mean difference (SMDs), or Cohen's *d*, its variance and standard error using formulae provided in Borenstein et al. (2009). An SMD is difference in means between the treatment and control groups divided by the pooled standard deviation of the outcome measure. Cohen's *d* can be biased in cases where sample sizes are small. Therefore, in all cases we will simply adjust *d* using Hedges’ method, adjusting Cohen's *d* to Hedges g using the following formula (Ellis, 2010):

$$g≅d(1-\frac{3}{4(n_{1}+n_{2})-9})$$

Formulas for effect size calculations will be used depending on data provided in included studies. For example, for studies reporting means (X) and pooled standard deviation (SD) for treatment (T) and control or comparison (C) at follow up only:

$$d=\frac{x_{Tp+1}-x_{Cp+1}}{SD}$$

If the study does not report the pooled standard deviation, it is possible to calculate it using the following formula:

$$SD_{p+1}=\sqrt{\frac{(n_{Tp+1}-1)\;SD_{Tp+1}^{2}+(n_{Cp+1}-1)SD_{Cp+1}^{2}}{n_{Tp+1}+n_{Cp+1}-2}}$$

Where the intervention is expected to change the standard deviation of the outcome variable, we will use the standard deviation of the control group only.

For studies reporting means ($\underline{X}$) and standard deviations (SD) for treatment and control or comparison groups at baseline (p) and follow up (p+1):

$$d=\frac{\Delta {\underline{X}}_{p+1}-\Delta {\underline{X}}_{p}}{SD_{p+1}}$$

For studies reporting mean differences ($\Delta \underline{X}$) between treatment and control and standard deviation (SD) at follow up (p+1):

$$d=\frac{\Delta {\underline{X}}_{p+1}}{SD_{p+1}}=\frac{{\underline{X}}_{Tp+1}-{\underline{X}}_{Cp+1}}{SD_{p+1}}$$

For studies reporting mean differences between treatment and control, standard error (SE) and sample size (n):

$$d=\frac{\Delta {\underline{X}}_{p+1}}{SE\sqrt{n}}$$

For studies reporting regression results, we will follow the approach suggested by Keef & Roberts (2004) using the regression coefficient and the pooled standard deviation of the outcome. Where the pooled standard deviation of the outcome is unavailable, we will use regression coefficients and standard errors or t‐statistics to do the following, where sample size information is available in each group:

$$d=t\sqrt{\frac{1}{nT}+\frac{1}{nC}}$$

where *n* denotes the sample size of treatment group and control. We will use the following where total sample size information (*N*) is available only (as suggested in Polanin, 2016):

$$d=\frac{2t}{\sqrt{N}}\;\;\;Var_{d}=\frac{4}{N}+\frac{d^{2}}{4N}$$

We will calculate the t‐statistic (*t*)by dividing the coefficient by the standard error. If the authors only report confidence intervals and no standard error we will calculate the standard error from the confidence intervals. If the study does not report the standard error, but report *t* we will extract and use this as reported by the authors. In cases in which significance levels are reported rather than t or se(b), then t will be imputed as follows:

Prob > 0.1: t=0.5
0.1 ≥ Prob > 0.05: t = 1.8
0.05 ≥ Prob > 0.01: t = 2.4
0.01 ≥ Prob: t = 2.8.

Where outcomes are reported in proportions of individuals, we will calculate the Cox‐transformed log odds ratio effect size (Sanchez‐Meca et al., 2003):

$$d=\frac{ln(OR)}{1.65}$$

where OR is the odds ratio calculated from the two‐by‐two frequency table.

Where outcomes are reported based on proportions of events or days, we will use the standardised proportion difference effect size:

$$d=\frac{p_{T}+p_{C}}{SD(p)}$$

where *p_t_* is the proportion in the treatment group and *p_c_* the proportion in the comparison group, and the denominator is given by:

$$SD(p)=\sqrt{p(1-p)}$$

where p is the weighted average of *p_c_* and *p_t_*:

$$p=\frac{nT\;pT+nC\;pc}{nT+nC}$$

In all cases after synthesis, we will convert pooled effect sizes to commonly used metrics such as percentage changes and mean differences in outcomes metrics typically used (e.g. weight in kg).

#### Dependent effect sizes

Estimation of a standard meta‐analytic effect size relies on the statistical assumption of independence of each included estimation of effect (Gleser & Olkin, 2007). Dependent effect sizes arise when one study provides multiple results for the same outcome of interest, when a study has multiple treatment arms compared to the same comparison group or multiple studies use the same dataset and report on the same outcome. We will therefore use the following rules to ensure that only statistically independent effect sizes are included in any one meta‐analysis. In general, we will only include one effect estimate per sample in a single meta‐analysis. However, if we identify 10 or more effect sizes for the same meta‐analysis, we will combine dependent effect sizes within the same meta‐analysis and use robust variance estimation (Tanner‐Smith & Tipton, 2014).

Where we identify several papers that report on the same study we will use effect sizes from the most recent publication. If we identify more than one study using the same data set, or where multiple outcomes are reported using similar outcome constructs within the same study, we will select the study or construct which is the most similar to other estimates for the same outcome type to enhance the potential for meta‐analysis. However, we will extract data and calculate effect sizes for the other outcome constructs. Where different studies report on the same programme, but use different samples (for example from different regions) we will include both estimates, treating them as independent samples, provided effect sizes are measured relative to separate control or comparison groups.

If one study reports multiple effect size estimates using different specifications for the same outcome, we will choose the one with the lowest risk of bias.

If studies report more than one follow up period for one outcome, we will identify the most common follow‐up period and include the follow up measures that match this most closely in the meta‐analysis. However, we will extract data and calculate effect sizes for all time points and report these in the review. If we identify studies with multiple treatment arms and only one comparison group, we will estimate an effect size for both arms, and either choose the effect estimate from the treatment arm that tests an intervention that most commonly resembles the other interventions included in the meta‐analysis to synthesise, or calculate a ‘synthetic effect’, as appropriate. Synthetic effects are calculated as the sample‐weighted average effect size, using appropriate formulae to recalculate variances according to Borenstein et al. (2009, chapter 24), making covariance assumptions as necessary.

Where studies report multiple effect sizes according to different follow‐up periods, we will synthesise differences in reported follow‐ups for those studies that did so statistically and narratively, but we will also calculate a synthetic effect size for any overarching analysis.

#### Unit of analysis

Unit of analysis errors can arise when the unit of allocation of a treatment is different to the unit of analysis of the study, and this is not accounted for in the analysis. We will assess studies for unit of analysis errors (The Campbell Collaboration, 2014). If unit of analysis errors exist, we will correct for this by adjusting the standard errors (Hedges, 2009, Higgins and Green, 2011, Waddington et al., 2012):

$$SE(d)\prime =SE(d)*\sqrt{1+(m-1)c}$$

where m is the average number of observations per cluster and c is the intra‐cluster correlation coefficient. Where included studies use robust Huber‐White standard errors to correct for clustering, we will calculate the standard error of *d* by dividing *d* by the t‐statistic on the coefficient of interest.

#### Missing data

In cases of missing or incomplete data, we will contact study authors to obtain the required information. If we are unable to obtain the necessary data, we will report the characteristics of the study but state that it could not be included in the meta‐analysis or reporting of effect sizes due to missing data.

### Methods of synthesis: Review questions 1, 2 and 3

Once we have identified all included studies we will map out all interventions, PITA characteristics, sectors and outcome measures provided in the included studies to determine how we will synthesise outcomes. We will only conduct meta‐analysis of studies which we assess to be sufficiently similar. The inclusion criteria for the review are broad and we anticipate including studies that report on a diverse set of interventions, sectors and outcomes. It is therefore difficult to predict how meta‐analysis will be used in the review prospectively. However, minimum criteria will be to only combine studies using meta‐analysis when we identify two or more effect sizes using a similar outcome construct and where the comparison group state is judged to be similar across the two, similar to the approach taken by Wilson et al. (2011). We provisionally suggest that we combine studies in the same bivariate analysis when they evaluate the same intervention type, for example information dissemination intervention or community based monitoring. We provisionally also suggest that we will attempt to run separate bivariate analyses by the PITA characteristics the intervention incorporates. Multivariate analyses can take into account multiple interventions and PITA characteristics as moderator variables, or examine the correlation between outcomes along the causal chain (mediator analysis). Hence, we will assess the extent to which we can model the heterogeneity in intervention and comparator using moderator analysis and meta‐regression. Where there are too few studies, or included studies are considered too heterogeneous in terms of interventions or outcomes, we will present a discussion of individual effect sizes along the causal chain. As heterogeneity exists in theory due to the variety of interventions and contexts included, we will use inverse‐variance weighted, random effects meta‐analytic models (Higgins & Green, 2011).

We will either use Stata's *metan* and *metareg* commands (Sterne et al., 2008), or the *metafor* package in R software to conduct the meta‐analysis (R Development Core Team, 2008; Viechtbauer, 2010).

We will conduct separate analyses for the major outcome groups:

- Service user / citizen (demand‐side behaviours).
- Service provider, public servant (supply‐side behaviours).
- Service delivery / access (quantity and quality).
- Service use.
- User satisfaction.
- Social and economic outcomes.

We will categorise outcomes that we identify into immediate outcomes (participation of service users and responses of service providers) under blocks 2 and 3 of the theory of change), intermediate outcomes (blocks 4 and 5) and endpoint stages (blocks 6, 7 and 8). We will subsequently analyse outcomes at each stage along the causal chain. We will also conduct moderator analysis to explore heterogeneity in the included studies. If feasible, we will use multiple meta‐regression to explore the association between the moderator variables and the outcomes of interest (Borenstein et al., 2009). We will use sub‐group analysis to explore heterogeneity by different treatment sub‐groups.

Where sufficient observations exist, we will undertake moderator analysis by the following groups of variables:

- Methodology: study design, risk of bias status, timing of evaluation (follow‐up length).
- Intervention characteristics: intervention, PITA characteristic, sector, co‐interventions (whether an intervention is implemented in isolation or as part of an integrated programme)ontext variables: region, country income level, democracy policy index score.
- Participant characteristics: e.g. sex, socio‐economic status.

We will also collect qualitative information from studies about the intervention and how engages with PITA characteristics. This information may subsequently be coded quantitatively to be used in moderator analysis, or used to classify intervention mechanisms in synthesis or in the further development of intervention causal chains. These characteristics may include: intervention objectives (to change processes, behaviours or both); whether interventions are strategic (complex, adaptable strategy to realise change) or tactical (tool‐based); the source of intervention (local, NGO, government or researcher‐led); the scale of the intervention (pilot experiment versus adoption of formal policy/law); use of demand side target group (e.g. households or citizen groups) and/or supply side (front line service delivery agents, local, regional or national service delivery managers, national government); agent on whom the onus for change is placed (unorganised citizens, community groups, NGO, government).

### Assessment of heterogeneity

We will assess heterogeneity by calculating the Q‐statistic, I^2^, and Tau^2^ to provide an estimate of the amount of variability in the distribution of the true effect sizes (Borenstein et al., 2009). We will complement this with assessment of heterogeneity of effect sizes graphically using forest plots. We will explore heterogeneity using moderator analysis in bivariate and, where possible, multivariate meta‐regression specifications.

#### Sensitivity analysis

We will conduct sensitivity analysis to assess whether the results of the meta‐analysis are sensitive to the removal of any single study. We will do this by removing studies from the meta‐analysis one‐by one and assessing changes in results. We will also assess sensitivity of results to inclusion of high risk of bias studies by removing these studies from the meta‐analysis and comparing results to the main meta‐analysis results.

#### Publication bias

We will attempt to reduce publication bias by searching for and including unpublished studies in the review. We will also undertake exploratory tests for the presence of publication bias through the use of contour‐enhanced funnel graphs (Peters et al., 2008) and statistical tests (Egger et al. 1997).

### Methods of synthesis: Review question 4

In the context of ‘real world’ programmes, we are often concerned about project design and implementation fidelity as the principal reasons why programme evaluations show limited impacts, which is partly why advocates of mixed‐methods evaluation approaches recommend collecting implementation process data (e.g. Bamberger et al., 2010; Snilstveit, 2012). We will use a thematic synthesis approach to extract information from project design and implementation documents and included impact studies on context, implementation and mechanisms. We will draw on the theory of change to do this as well as key frameworks for understanding why governance change may or may not happen, such as the Kaleidoscope Model (Resnick et al, 2015), which identifies key determinants of political change that could be influenced by PITA interventions, such as the relative power of proponents versus opponents in the adoption phase of the policy cycle. Finally, these will be compared with the effect sizes for each study to attempt to draw some conclusions about the extent to which the identified themes are systematically associated with outcomes.

Realist synthesis highlights variation in programme design in explaining differences in outcomes across contexts (Pawson, 2006). Realists argue that the effectiveness of a programme depends on the combined action of the behavioural mechanisms underlying it and the context in which it takes place. Behavioural mechanisms operate through the values, beliefs and past experiences of individuals in the social system. Thus, factors such as interpersonal networks and individual agency are important in the adoption and rejection of an intervention. The action of mechanisms depends in part on the context in which they are used. Behaviour change is achieved via the entire system of social relationships (the context) and, therefore, an intervention geared towards the achievement of behaviour change must be aligned with the context in which it is used (Waddington et al., 2009). The approach that draws these concepts together is called context‐mechanism‐outcome (CMO) synthesis. There are different ways of conducting CMO synthesis including iterations of a causal model (e.g. theory of change diagram) (Waddington et al., 2014), CMO tables (Petrosino et al., 2012) and qualitative comparative analysis (QCA) (Ton et al., 2017). QCA articulates the associations between empirical effects and context and mechanism conditions drawing on “truth‐tables” which articulate all possible instances of conditions and show which cases share the same combination of conditions.

As CMO is largely an iterative process, the full list of CMO codes for analysis will be developed through the synthesis. Potential themes and context factors for investigation include: the enabling conditions that allow for project success; and systemic and social levels targeted by the intervention. Key enabling conditions could include whether the intervention is designed to build off of and work within local systems of power relations and social norms that uphold the social contract between the State and society (as in Halloran's “accountability ecosystem”, 2015), or the political salience of the public service targeted (Mcloughlin and Batley, 2012). Where key enabling conditions are already in place, an intervention effectively designed may be successfully implemented in isolation; where key conditions are missing, the intervention design may need to be adjusted or expanded to include complementary interventions that seek to strengthen the enabling environment. For example, an intervention seeking to build transparency and accountability through open data interventions may need to build a coalition of support that engages people at the point in the system targeted for data release, upstream, downstream, and externally to create an environment in which data is provided, demanded, and used (Hogge, 2010). These enabling conditions may change depending on context factors such as the target level of the intervention‐ whether it targets service delivery at community, sub‐national, or national level (E‐Pact Consortium, 2016) or whether the external stakeholders it seeks to engage are organised civil society or interest groups, marginalised or vulnerable groups, or citizens and service users more broadly (McGee and Gaventa, 2010). We also plan to conduct more detailed analysis of whether the bottleneck for good governance is likely to be properly identified as resting with citizens (e.g. lack of organization, lack of knowledge/capacity), with the system (e.g. lack of opportunities for citizens to engage), or with individual service providers (e.g. power relations, corruption). Data will be collected qualitatively from studies and then grouped using thematic synthesis or content analysis, and presented in tabular format.

Van der Knapp et al. (2006) is possibly the first example of a systematic review that explicitly incorporates context‐mechanism‐outcome synthesis. These authors indicate that the CMO synthesis is undertaken after the systematic review and meta‐analysis. The broad approach is as follows:

- Information on possible programme mechanisms is collected from studies during the coding phase. Van der Knapp indicate that one way of doing this is to search included studies for information about how or why the intervention is supposed to work, stating that “The focus in such a classification can be on behavioral and social ‘cogs and wheels’ of the intervention… but could also include administrative or legal mechanisms.” (p.6). The review team also anticipates identifying and coding mechanisms associated with particular interventions or PITA.
- Information on contextual factors will also be collected during the coding phase. This is partly contained in the detailed information about the comparison condition, co‐interventions and background information about participants collected from included studies and project and programme design and implementation documents, as well as any contextual information collected from international datasets.

We will explore appropriate methods to link the meta‐analysis with context and mechanism information, such as by using QCA (Befani, 2016). We note that the application of QCA is limited by the number of included studies, their comparability and the completeness of reporting within them, hence the application of QCA might be difficult in this review. We therefore also plan to use tabular reporting by intervention and further iterations of the theory of change by PITA groups.

### Methods of synthesis: Review question 5

We will draw on standard approaches to synthesise economic appraisal evidence depending on the available cost evidence (Shemilt et al., 2011; Shemilt et al., 2008). If we are unable to identify sufficient data to calculate cost‐effectiveness ratios for example, we will simply report the cost data that we identify in tables alongside effect sizes.

## Review authors

**Lead review author:** The lead author is the person who develops and co‐ordinates the review team, discusses and assigns roles for individual members of the review team, liaises with the editorial base and takes responsibility for the on‐going updates of the review.

**Table:** 

Name: Hugh Waddington
Title: Senior Evaluation Specialist
Affiliation: International Initiative for Impact Evaluation
Address: 36 Gordon Square
City, State, Province or County: London
Post code: WC1H 0PD
Country: U.K.
Phone: +44 207 958 8351
Email: hwaddington@3ieimpact.org
**Co‐author(s):**
Name: Ada Sonnenfeld
Title: Research Consultant, 3ie
Country: U.K.
Email: asonnenfeld@3ieimpact.org
Name: Marie Gaarder
Title: Head of Evaluation, 3ie
Country: Norway
Email: mgaarder@3ieimpact.org

## Roles and responsibilities

- **Content:** All members of the review team have substantive expertise in a range of topics in international development including interventions for project and programme governance. Jennifer Stevenson (JS) was a member of the team that produced an evidence gap map on interventions to improve state‐society relations, the results of which will be used to produce this systematic review, and also led the work on community‐based monitoring in a systematic review of education interventions (Snilstveit et al., 2015). Ada Sonnenfeld (AS), Marie Gaarder (MG) and HJW have field experience in development policy, governance programme design implementation, monitoring and evaluation. Hugh Waddington (HJW) contributed to a forthcoming review on community‐driven development (White et al., 2018). The team will be supported by an advisory group of academics and policy makers with specific expertise in governance.
- **Systematic review methods:** Together, HJW, JS and MG have substantial expertise in systematic review methodology. HJW is a Co‐Chair and Editor of the International Development Coordinating Group (IDCG) of the Campbell Collaboration and has authored, edited, peer reviewed and supported over 100 systematic reviews on international development topics. JS has also designed, worked on, and supported, a number of systematic reviews and meta‐analyses. MG led the systematic reviews team at the World Bank's Independent Evaluation Group and originated the evidence gap map approach.
- **Statistical analysis**: HJW and JS have substantial experience in statistical analysis and meta‐analysis for systematic reviews and will lead the work on calculation of effect sizes and statistical meta‐analysis.
- **Information retrieval:** JS will lead the work on the development of the search strategy. She has experience with systematic searching as part of several systematic reviews and evidence gap maps. The team will be supported by John Eyers, an information specialist with over 20 years’ experience, who has supported the development of search strategies for a large number of systematic reviews in the fields of international development, nutrition, public health and health care.

## Sources of support

We are grateful for financial support from the United States Agency for International Development (USAID) via NORC project number 7554.070.01.

We are also grateful to Rohini Pande for advice, the two anonymous referees and the stakeholder advisory review group for their helpful inputs:

Andrew Greer, USAID

Annette Brown, FHI360

Courtney Tolmey, Results for Development

Erik Wibbels, USAID

Guy Grossman, University of Pennsylvania

Joanne Trotter, Aga Khan Foundation

Laura Adams, USAID

Morgan Holmes, USAID.

## Declarations of interest

None of the team members have any financial interests in the review or have worked on primary research covering the interventions covered by the review.

## Preliminary timeframe

- Planned date of submission of draft review: August 2018.
- Planned date of completion of the review: September 2018.

## Plans for updating the review

The authors will undertake, or contribute to, updates once resources are identified and further high quality studies become available.

## AUTHOR DECLARATION

### Authors’ responsibilities

By completing this form, you accept responsibility for preparing, maintaining and updating the review in accordance with Campbell Collaboration policy. The Campbell Collaboration will provide as much support as possible to assist with the preparation of the review.

A draft review must be submitted to the relevant Coordinating Group within two years of protocol publication. If drafts are not submitted before the agreed deadlines, or if we are unable to contact you for an extended period, the relevant Coordinating Group has the right to de‐register the title or transfer the title to alternative authors. The Coordinating Group also has the right to de‐register or transfer the title if it does not meet the standards of the Coordinating Group and/or the Campbell Collaboration.

You accept responsibility for maintaining the review in light of new evidence, comments and criticisms, and other developments, and updating the review at least once every five years, or, if requested, transferring responsibility for maintaining the review to others as agreed with the Coordinating Group.

### Publication in the Campbell Library

The support of the Coordinating Group in preparing your review is conditional upon your agreement to publish the protocol, finished review, and subsequent updates in the Campbell Library. The Campbell Collaboration places no restrictions on publication of the findings of a Campbell systematic review in a more abbreviated form as a journal article either before or after the publication of the monograph version in Campbell Systematic Reviews. Some journals, however, have restrictions that preclude publication of findings that have been, or will be, reported elsewhere and authors considering publication in such a journal should be aware of possible conflict with publication of the monograph version in Campbell Systematic Reviews. Publication in a journal after publication or in press status in Campbell Systematic Reviews should acknowledge the Campbell version and include a citation to it. Note that systematic reviews published in Campbell Systematic Reviews and co‐registered with the Cochrane Collaboration may have additional requirements or restrictions for co‐publication. Review authors accept responsibility for meeting any co‐publication requirements.

**I understand the commitment required to undertake a Campbell review, and agree to publish in the Campbell Library. Signed on behalf of the authors**:

**Form completed by: Hugh Waddington**

**Date: 22 February 2017**

## Footnotes

1 Killias et al. (2016) are registering a review on the effectiveness of anti‐corruption measures. The study protocol is available from the Campbell library: https://www.campbellcollaboration.org/media/k2/attachments/Killias_Corruption_Protocol.pdf.

2 For an example, see Doocy and Tappis (2017).

## Example search strategy for Web of Science (Social Sciences Citation Index)

#26 AND #11 AND #5

# 28

#27 AND #26 AND #5

# 27

TS=(“random* control* trial*” or “random* trial*” or RCT or “propensity score matching” or PSM or “regression discontinuity design” or RDD or “difference in difference*” or DID or difference‐in‐difference or evaluat* or matching or “interrupted time series” or (random* NEAR/3 allocat*) or “instrumental variable*” or IV or ((quantitative or “comparison group” or counterfactual or “counter factual” or counter‐factual or experiment* or quasi‐experimental or “quasi experimental”) NEAR/3 (design or study or analysis)) or QED or “field experiment” or “field trial”)

# 26

#25 OR #24 OR #23 OR #22 OR #21 OR #20 OR #19 OR #18 OR #17 OR #16 OR #15 OR #14 OR #13 OR #12

# 25

TS=(((sms OR “short message*” OR “text message*” OR bulk‐messag* OR “bulk messag*” OR mass‐messag* OR “mass messag*” OR “public awareness” OR engagement OR information) NEAR/3 (campaign* OR strategy OR strategies)) OR “information dissemination”)

# 24

TS=(“standard service*” OR standard‐service* OR standardized‐service* OR standardised‐service* OR “standardized service*” OR “standardised service*”)

# 23

TS=((service* OR one‐stop OR “one stop”) NEAR/1 (centre* OR center* OR shop*)) OR TS=((communit* OR community‐based) NEAR/3 monitor*)

# 22

TS=((“social* accountab*”) NEAR/2 (mechanism* OR system* OR arrange* OR organi* OR regulat*)) OR TS=((social OR public) NEAR/1 audit)

# 21

TS=(“report card*” OR reportcard* OR report‐card* OR “score card*” OR scorecard* OR score‐card*) OR TS=(“political reserv*” OR “reserved place*” OR “reserved position*” OR “reserved seat*”)

# 20

TS=(“e governance” OR e‐governance OR egovernance OR “electronic governance”) OR TS=((politic* NEAR/3 (inclus* OR participat* OR quota OR quotas)) OR (quota* NEAR/3 participat*))

# 19

TS=((((disaster* OR disaster‐risk*) NEAR/6 (respond* OR response* OR management OR reduc* OR preparedness)) OR DRR) NEAR/3 (committee* OR council* OR association* OR shura))

# 18

TS=((communit* OR district* OR cluster OR cluster‐level) NEAR/6 development NEAR/3 (committee* OR council* OR association* OR shura))

# 17

TS=((“natural resource*” OR natural‐resource* OR NRM OR “common property” OR common‐property OR “common resource*” OR common‐resource* OR water‐use* OR “water use*” OR “water management” OR water‐management OR land‐use* OR “land use*” OR land‐management OR “land management” OR irrigat*) NEAR/6 (participat* OR transparen* OR inclus* OR represent* OR consult* OR community* OR committee* OR council* OR association* OR group* OR shura))

# 16

TS=((health OR healthcare OR hospital*) NEAR/3 (committee* OR “action group*” OR council* OR association* OR shura))

# 15

TS=((inclus* OR particip*) NEAR/6 (strateg* OR action* OR budget* OR development OR plan*))

# 14

TS=(“community engagement” OR “community consultation*” OR (civic NEAR/3 education))

# 13

TS=((communit* OR inclus* OR particip*) NEAR/6 (((climate‐change OR “climate change”) NEAR/2 (adapt* OR mitigat* OR vulnerab*)) or resilien*))

# 12

TS=(((disaster* NEAR/2 reduc* NEAR/2 risk*) OR (disaster* NEAR/2 (respond* OR response* OR manag*)) OR ((hazard* OR risk* OR vulnerab*) NEAR/2 (map* OR assess*)) OR HVA OR HVRA OR DRR) NEAR/6 (participat* OR inclus* OR consult* OR communit*))

# 11

#10 OR #9 OR #8 OR #7 OR #6

# 10

TS=(economic NEAR/2 model*)

# 9

TS=(“cost minimi*” OR “cost‐utilit*” OR “health utilit*” OR “economic evaluation*” OR “economic review*” OR “cost outcome” OR “cost analys*” OR “economic analys*” OR “budget* impact analys*”)

# 8

TS=(cost‐effective* OR cost‐benefit OR costs)

# 7

TS=(“life year” OR “life years” OR qaly* OR daly*)

# 6

TS=((cost OR economic*) AND (costs OR cost‐effectiveness OR markov))

# 5

#4 OR #3 OR #2 OR #1

# 4

TS=((lmic or lmics or “third world” or “lami countr*”)) OR TS=(“transitional countr*”)

# 3

TS=(((developing or “less* developed” or “under developed” or underdeveloped or “middle income” or “low* income”) NEAR/1 (economy or economies))) OR TS=((low* NEAR/1 (gdp or gnp or “gross domestic” or “gross national”))) OR TS=((low NEAR/3 middle NEAR/3 countr*))

# 2

TS=(“Developing Countries”) OR TS=(Africa or Asia or Caribbean or “West Indies” or “South America” or “Latin America” or “Central America”) OR TS=(((developing or “less* developed” or “under developed” or underdeveloped or “middle income” or “low* income” or underserved or “under served” or deprived or poor*) NEAR/1 (countr* or nation* or population* or world)))

# 1

TS=((Afghanistan or Albania or Algeria or Angola or Argentina or Armenia or Armenian or Aruba or Azerbaijan or Bangladesh or Benin or Byelarus or Byelorussian or Belarus or Belorussian or Belorussia or Belize or Bhutan or Bolivia or Bosnia or Herzegovina or Hercegovina or Botswana or Brasil or Brazil or Bulgaria or “Burkina Faso” or “Burkina Fasso” or “Upper Volta” or Burundi or Urundi or Cambodia or “Khmer Republic” or Kampuchea or Cameroon or Cameroons or Cameron or Camerons or “Cape Verde” or “Central African Republic” or Chad or China or Colombia or Comoros or “Comoro Islands” or Comores or Mayotte or Congo or Zaire or “Costa Rica*” or “Cote d'Ivoire” or “Ivory Coast” or Cuba or Djibouti or “French Somaliland” or Dominica or “Dominican Republic” or “East Timor” or “East Timur” or “Timor Leste” or Ecuador or Egypt or “United Arab Republic” or “El Salvador” or Eritrea or Ethiopia or Fiji or Gabon or “Gabonese Republic” or Gambia or Gaza or “Georgia Republic” or “Georgian Republic” or Ghana or Grenada or Guatemala or Guinea or Guiana or Guyana or Haiti or Hungary or Honduras or India or Maldives or Indonesia or Iran or Iraq or Jamaica or Jordan or Kazakhstan or Kazakh or Kenya or Kiribati or Korea or Kosovo or Kyrgyzstan or Kirghizia or “Kyrgyz Republic” or Kirghiz or Kirgizstan or “Lao PDR” or Laos or Lebanon or Lesotho or Basutoland or Liberia or Libya or Macedonia or Madagascar or “Malagasy Republic” or Malaysia or Malaya or Malay or Sabah or Sarawak or Malawi or Mali or “Marshall Islands” or Mauritania or Mauritius or “Agalega Islands” or Mexico or Micronesia or “Middle East” or Moldova or Moldovia or Moldovian or Mongolia or Montenegro or Morocco or Ifni or Mozambique or Myanmar or Myanma or Burma or Namibia or Nepal or “Netherlands Antilles” or “New Caledonia” or Nicaragua or Niger or Nigeria or Pakistan or Palau or Palestine or Panama or Paraguay or Peru or Philippines or Philipines or Phillipines or Phillippines or “Puerto Ric*” or Romania or Rumania or Roumania or Rwanda or Ruanda or “Saint Lucia” or “St Lucia” or “Saint Vincent” or “St Vincent” or Grenadines or Samoa or “Samoan Islands” or “Navigator Island” or “Navigator Islands” or “Sao Tome” or Senegal or Serbia or Montenegro or Seychelles or “Sierra Leone” or “Sri Lanka” or “Solomon Islands” or Somalia or “South Africa” or Sudan or Suriname or Surinam or Swaziland or Syria or Tajikistan or Tadzhikistan or Tadjikistan or Tadzhik or Tanzania or Thailand or Togo or Togolese Republic or Tonga or Tunisia or Turkey or Turkmenistan or Turkmen or Uganda or Ukraine or Uzbekistan or Uzbek or Vanuatu or “New Hebrides” or Venezuela or Vietnam or “Viet Nam” or “West Bank” or Yemen or Yugoslavia or Zambia or Zimbabwe) NOT (“African‐American*” OR “African‐American*” OR “Mexican American*” OR “American Indian*” OR “Asian American*” OR “native american*”))

**Example search strategy for health databases**

**Ovid MEDLINE**

1 ((Afghanistan or Albania or Algeria or Angola or Argentina or Armenia or Armenian or Aruba or Azerbaijan or Bangladesh or Benin or Byelarus or Byelorussian or Belarus or Belorussian or Belorussia or Belize or Bhutan or Bolivia or Bosnia or Herzegovina or Hercegovina or Botswana or Brasil or Brazil or Bulgaria or “Burkina Faso” or “Burkina Fasso” or “Upper Volta” or Burundi or Urundi or Cambodia or “Khmer Republic” or Kampuchea or Cameroon or Cameroons or Cameron or Camerons or “Cape Verde” or “Central African Republic” or Chad or China or Colombia or Comoros or “Comoro Islands” or Comores or Mayotte or Congo or Zaire or “Costa Rica*” or “Cote d'Ivoire” or “Ivory Coast” or Cuba or Djibouti or “French Somaliland” or Dominica or “Dominican Republic” or “East Timor” or “East Timur” or “Timor Leste” or Ecuador or Egypt or “United Arab Republic” or “El Salvador” or Eritrea or Ethiopia or Fiji or Gabon or “Gabonese Republic” or Gambia or Gaza or “Georgia Republic” or “Georgian Republic” or Ghana or Grenada or Guatemala or Guinea or Guiana or Guyana or Haiti or Hungary or Honduras or India or Maldives or Indonesia or Iran or Iraq or Jamaica or Jordan or Kazakhstan or Kazakh or Kenya or Kiribati or Korea or Kosovo or Kyrgyzstan or Kirghizia or “Kyrgyz Republic” or Kirghiz or Kirgizstan or “Lao PDR” or Laos or Lebanon or Lesotho or Basutoland or Liberia or Libya or Macedonia or Madagascar or “Malagasy Republic” or Malaysia or Malaya or Malay or Sabah or Sarawak or Malawi or Mali or “Marshall Islands” or Mauritania or Mauritius or “Agalega Islands” or Mexico or Micronesia or “Middle East” or Moldova or Moldovia or Moldovian or Mongolia or Montenegro or Morocco or Ifni or Mozambique or Myanmar or Myanma or Burma or Namibia or Nepal or “Netherlands Antilles” or “New Caledonia” or Nicaragua or Niger or Nigeria or Pakistan or Palau or Palestine or Panama or Paraguay or Peru or Philippines or Philipines or Phillipines or Phillippines or “Puerto Ric*” or Romania or Rumania or Roumania or Rwanda or Ruanda or “Saint Lucia” or “St Lucia” or “Saint Vincent” or “St Vincent” or Grenadines or Samoa or “Samoan Islands” or “Navigator Island” or “Navigator Islands” or “Sao Tome” or Senegal or Serbia or Montenegro or Seychelles or “Sierra Leone” or “Sri Lanka” or “Solomon Islands” or Somalia or “South Africa” or Sudan or Suriname or Surinam or Swaziland or Syria or Tajikistan or Tadzhikistan or Tadjikistan or Tadzhik or Tanzania or Thailand or Togo or Togolese Republic or Tonga or Tunisia or Turkey or Turkmenistan or Turkmen or Uganda or Ukraine or Uzbekistan or Uzbek or Vanuatu or “New Hebrides” or Venezuela or Vietnam or “Viet Nam” or “West Bank” or Yemen or Yugoslavia or Zambia or Zimbabwe) not (“African‐American*” or “African‐American*” or “Mexican American*” or “American Indian*” or “Asian American*” or “native american*”)).ti,ab,hw.

2 (“Developing Countries” or Africa or Asia or Caribbean or “West Indies” or “South America” or “Latin America” or “Central America” or ((developing or “less* developed” or “under developed” or underdeveloped or “middle income” or “low* income” or underserved or “under served” or deprived or poor*) adj1 (countr* or nation* or population* or world))).ti,ab,hw,kw.

3 (((developing or “less* developed” or “under developed” or underdeveloped or “middle income” or “low* income”) adj1 (economy or economies)) or (low* adj1 (gdp or gnp or “gross domestic” or “gross national”)) or (low adj3 middle adj3 countr*)).ti,ab,hw,kw. (9725)

4 (lmic or lmics or “third world” or “lami countr*” or “transitional countr*”).ti,ab,hw,kw. (5281)

5 or/1‐4

6 (“random* control* trial*” or “random* trial*” or RCT or “propensity score matching” or PSM or “regression discontinuity design” or RDD or “difference in difference*” or DID or difference‐in‐difference or evaluat* or matching or “interrupted time series” or (random* adj3 allocat*) or “instrumental variable*” or IV or ((quantitative or “comparison group” or counterfactual or “counter factual” or counter‐factual or experiment* or quasi‐experimental or “quasi experimental”) adj3 (design or study or analysis)) or QED or “field experiment” or “field trial”).ti,ab,hw,kw.

7 exp Randomized Controlled Trial/ or Randomized Controlled Trials as Topic/ or random allocation/ or Propensity Score/ or Quasi‐Experimental Studies/ or Controlled Before‐After Studies/ or Interrupted Time Series Analysis/

8 6 or 7

9 (((communit* or village* or stakeholder*) adj3 (engag* or consult* or meeting* or outreach* or represent* or participat* or network)) or (civic adj3 education) or audit or “social responsibility” or (moral* adj2 obligat*)).ti,ab,hw,kw.

10 Community Participation/ or Community Networks/ or Stakeholder Participation/ or Social responsibility/ or Moral Obligations/ or Management Audit/

11 ((inclus* or participat*) adj6 (strateg* or action* or budget* or plan*)).ti,ab,hw,kw.

12 ((health or healthcare or hospital* or women* or communit*) adj3 (committee* or “action group*” or council* or association* or shura)).ti,ab,hw,kw.

13 ((((disaster* or disaster‐risk*) adj6 (respond* or response* or management or reduc* or preparedness)) or DRR) adj3 (committee* or council* or association* or shura)).ti,ab,hw,kw.

14 (“e governance” or e‐governance or egovernance or “electronic governance”).ti,ab,hw,kw.

15 (“report card*” or reportcard* or report‐card* or “score card*” or scorecard* or score‐card*).ti,ab,hw,kw.

16 ((accountab* adj2 (mechanism* or system* or arrange* or organi* or regulat*)) or ((social or public) adj1 audit) or ((communit* or community‐based) adj3 monitor*)).ti,ab,hw,kw.

17 ((service* or one‐stop or “one stop”) adj1 (centre* or center* or shop*)).ti,ab,hw,kw.

18 (“standard service*” or standard‐service* or standardized‐service* or standardised‐service* or “standardized service*” or “standardised service*”).ti,ab,hw,kw.

19 Mass Media/ or Electronic Mail/ or Internet/ or Text Messaging/ or Communication/ or Health promotion/ or Consumer Health Information/ or Information Dissemination/

20 ((((sms or “short message*” or “text message*” or bulk‐messag* or “bulk messag*” or mass‐messag* or “mass messag*” or “public awareness” or engagement or information or “mass media” or email or e‐mail or “electronic mail” or internet or communicat*) adj3 (campaign* or strategy or strategies)) or “health promot*” or “consumer health information” or (information* adj2 disseminat*)) and (entitle* or rights or (health adj1 (service* or provider*) adj3 performance*) or “service provision”)).ti,ab,hw,kw

21 or/9‐20

22 5 and 8 and 21

23 limit 22 to yr=″2000 ‐Current*

24 exp health facilities/ or Delivery of Health Care/ or Regional Health Planning/

25 (hospital or hospitals or infirmary or infirmaries or clinic or clinics or ((health or medical) adj (centre* or center* or facilit*)) or (deliver* adj2 (“health care” or healthcare or “health service*”)) or (plan* adj2 region* adj2 health)).ti,ab,hw,kw.

26 24 or 25

27 23 and 26

## Examples of included and excluded interventions

**Table:**

**Include**	**Exclude**	**Rationale**
Intervention: Second Northern Uganda Social Action Fund (Fiala and Premand, 2017) Country: Uganda PITA: A Summary: This evaluation looks at a social‐accountability component that was added on to the standard CDD program (P), wherein all studied communities were participants in the CDD program, and the differentiation came from random allocation to two types of accountability‐focused add‐on interventions (A). Because only the add‐on intervention is evaluated, this study is classed as accountability (A) focused.	Intervention: National Solidarity Program (NSP) (Beath et al., 2012) Country: Afghanistan PITA: P, I Summary: Though the intervention aims to improve the capacity of communities to participate in the prioritisation and management of public service provision (P), including specific requirements to engage women (I), the cornerstone of the intervention is a large (ave. USD $30,000) block grant for community infrastructure, which comparison communities do not receive. Thus, because they do not receive the same access to services as intervention communities, it is impossible to isolate the value‐add of the PITA elements of the intervention.	While these are both CDD interventions, they differ in the type of comparator group. In Uganda, the comparator communities still participate in the CDD intervention. In Afghanistan, they do not receive the CDD block grant, which makes isolating the value‐add of the PITA interventions challenging, because they are tied so closely to the block grant implementation.
Intervention:*Tuungane*(Humphreys et al., 2012) Country: Democratic Republic of Congo (DRC) PITA: P, I Summary: The *Tuungane* evaluation measures the impact of the social mobilisation interventions of this CDR project through an experiment in which both treatment and control communities receive a small grant, and their inclusive decision‐making capacities are evaluated (P). Further, intervention communities were randomly assigned to require gender parity in decision‐making groups or not, and thus the value‐add of quotas for women's participation can be isolated (I). In this case, the quotas ensure women citizens are able to contribute to community decision‐making, on par with male citizens.	Intervention: Mandated political representation for women (Iyer et al., 2011) Country: India PITA: I Summary: This paper looks at the impact of introducing quotas for women's participation in local government councils (I). However, these are elected positions wherein the incumbents are formal government employees. Thus, while such a change may impact women citizen's access to public officials, it does not create specific opportunities for private citizens to engage with public officials.	Both these interventions incorporate quotas to ensure women's participation. However, in DRC the intervention creates quotas for women's participation *in an external citizen engagement intervention*, whereas the Iyer et al. 2011 study targets the “I” characteristics of the formal political system, which is not the governance domain of focus for this review.
Intervention: Voter Opinion and Involvement through Citizen Education (VOICE) (Finkel, 2012) Country: Democratic Republic of Congo PITA: T Summary: Though the title of the intervention focuses on the word “voter,” the intervention actually comprises a series of efforts to improve citizens’ knowledge of the decentralisation process, and how they could engage with it to make it more participatory. Outcomes measured include knowledge of political processes and opportunities for engagement (going beyond voting) and changes in practices of engaging with the decentralisation process.	Intervention: Corruption and public expenditure campaign (Chong et al. 2012) Country: Mexico PITA: T Summary: While this experiment hinges on providing information about corruption and public expenditure of public servants to their constituents, the outcomes measured are restricted to the intervention's impact on the subsequent election.	Both of these interventions focus on transparency. However, the Chong et al. 2012 study only measures impacts on voting, which fall within the political systems governance domain, and thus outside the scope of this review. By contrast, because the Finkel 2012 study measures a more diverse set of outcomes that look at participation in non‐electoral processes, it is includable.
Intervention: Joint Forestry Management (Persha and Meshak, 2016) Country: Tanzania PITA: P Summary: This intervention devolves control over common resource management completely, from the government to communities (P). Thus, communities are empowered to create their own rules for natural resource use, and they share accountability with the government for the enforcement of those rules.	Intervention: Decentralisation of Water Supply (Asthana, 2012) Country: India PITA: T Summary: This study evaluates the impact of decentralisation from state‐level government to local government. Thus, though the intervention was designed to reduce corruption, it does not engage citizens in the process or create specific opportunities for them to engage.	Though both cases focus on the management of common‐good natural resources, and both look at the impact of decentralisation, the India study only devolves power from one level of government to a lower level, and thus resides within the sphere of internal systems management, as citizens are not engaged in the process. The Tanzania study, in contrast, empowers communities to create their own rules for managing natural resources, which may differ from state‐level rules.
Intervention: Citizen Report Cards (Bjorkman et al., 2006) Country: Uganda PITA: T, A Summary: This study looks at the impacts of an intervention in which “report cards” of health service provision were disseminated amongst communities (T), and a series of interface meetings between service providers and citizens were organised to review the reports and identify an action plan for improvements (A).	Intervention: MIRA Makwanpur (Manandhar et al., 2004) Country: Nepal PITA: P Summary: This intervention formed community‐based, participatory women's health groups with the aim of identifying key local challenges and potential solutions (P), with the ultimate goal of improving birth outcomes.	Though both of these health‐sector interventions work to identify challenges and develop action plans to improve outcomes, the Uganda study enables citizens to hold public health providers accountable for delivering services, and jointly develops strategies for improvement to which the health providers are accountable. In the Nepal example, the women's groups are empowered to take responsibility for their own healthy practices; there are no requirements on the health service providers to take responsibility for addressing challenges the women identify. The intervention aims to change health outcomes *outside* the sphere of public service delivery.
Intervention: Raskin subsidy identification cards (Banerjee et al., 2016) Country: Indonesia PITA: T Summary: This study presents the results of an experiment in which recipients of the Raskin food subsidy were sent cards confirming their right to the subsidy; an alternative intervention in which lists of eligible households in communities were publicly displayed; and a control set where there were no changes in publication of eligibility for the subsidy. The aim was to test the effect of these different transparency initiatives on reducing corruption in subsidy provision.	Intervention: Ciudad Mujer (Women's City) (Bustelo et al., 2016) Country: El Salvador PITA: T Summary: This intervention created “one stop shops” for a variety of public services targeted to women, under the auspices of a health facility. When women arrived, they would take part in an orientation that explained all of the different services they could access at the facility, improving their knowledge of their rights to services (T).	Both of these interventions aim to increase citizens’ knowledge of their rights to access services (or public subsidies). However, the intervention in El Salvador was purely about access to services; it did not aim to change the way that women engaged with public service providers, except to encourage them to take advantage of the services. In contrast, the experiment in Indonesia had the explicit aim of attempting to reduce corruption in the subsidy program by limiting service providers’ ability to direct who received the subsidy and who didn't. Thus, in this latter case, the change in knowledge changes the power relations between service provider and user.

## Coding tools

*Note: this coding tool may be adapted following further piloting*.

**Descriptive / qualitative coding tool**

**Table:**

	**Description**	**Question**	**Coding**
**Report identification**	**Unique study identification #**		**For example, PITA001**
	**First author ‐ impact evaluation**	**Surname**	**Surname**
	**Other papers used for coding**	**First author surname and type of paper of any qualitative, descriptive quantitative, process evaluations, used for coding**	
	**Publication date**	**Year (letter ‐ if more than one study from that author and that year)**	**XXXX (a)**
	**Publication type**	**What is the impact evaluation publication type?**	1= Peer‐reviewed journal 2= Book chapter/book 3= Conference paper 4= Organisation report 5= Working paper 6= Implementation document 7= other grey 8= PhD thesis / dissertation
	**Funding agency**	**Who is funding the evaluation/study?**	**1= Public institution (e.g. govt, university, research institute)** 2= Private institution (e.g. private company) 3= Multilateral Organisation (World Bank, UN) 4 = Foundations 5 = NGO 8= Not clear 9= Not applicable (Non‐funded)
	**Name of funding agency**	**Please add name of the agency funding the evaluation**	**Open answer**
	**Independence of evaluation**	**What level of independence is there between the implementing agency and study team?**	**1=Funding and author team independent of implementers/ funders of programme** **2=Funding independent of implementers/ funders of programme, but includes authors from funder/ implementer** **3=Evaluation funded and undertaken by funders/ implementers** **8=Unclear**
	**Independent data collection**	**Has the data been collected by an independent party?**	**1= Yes 2=No 8=Not clear**
	**Conflict of interest**	**Is there a potential conflict of interest associated with study which could influence results collected/reported? (eg. Is there a declaration of conflict of interest? Is any of the authors related in any way to the funding or implementing institution?)**	**1=Yes 2=No 8=Not clear**
	**Comments on conflict of interest**	**Please add reason for your answer to whether there is a conflict of interest.**	**Open answer**
	**Language of publication**	**Language of publication of the impact evaluation, e.g. Spanish, English etc.**	**Open answer**
	**Other methods**	**If the impact evaluation addresses other questions than effectiveness note questions and methods used here.**	**Open answer (this will include for example mixed‐methods to assess implementation, adherence, participant views etc)**
**Context**	**Country**	**List countries the study was conducted in**	**Country 1, Country 2, etc.**
	**Detailed location**	**If provided, give detailed information on where the study took place within a country, for example regions/districts covered**	**Open answer**
	**World Bank Region**	**Select region(s) the study was conducted in according to World Bank. For more info on region classification see** http://data.worldbank.org/country	**1= East Asia & Pacific** **2= Europe & Central Asia** **3= Latin America & Caribbean** **4= Middle East & North Africa** **5=South Asia** **6=Sub‐Saharan Africa**
	**WB Income category**	**Select the World Bank income classification of the country at the time of the study**	**1 = Low income country** **2 = Lower‐middle income country** **3 = Upper‐middle income country**
	**Country performance ‐ governance indicators**	**Report the country performance on the indicator in the year of**	
	**Baseline socio‐economic status of participants**	**Report any data / description on baseline socio‐economic status of participants**	**Open answer**
**Intervention descriptives**	**Programme or project name**	**State the programme or project name. If no name, then list the location**	**Open answer**
	**Intervention type**	**If intervention is PITA specific, please indicate which type of PITA‐specific intervention**	**1 = Information campaigns** **2 = Standardised service delivery boards** **3 = One‐stop service centres** **4 = Community‐based monitoring** **5 = Social accountability mechanism or system** **6 = Social audit or public audit** **7 = Report‐cards or scorecards** **8 = Reserved seats or quotas for participation** **9 = e‐Governance** **10 = Community‐based public service committees** **11= Participatory budgeting** **12 = Participatory development planning** **13 = Inclusive strategy development** **14 = Community consultation** **15 = Civic education** **16 = Community‐based climate change adaptation** **17 = Participatory hazard and vulnerability mapping or risk assessments**
	**PITA characteristics**	**Indicate which type of characteristics targeted by the intervention (multiple choice ‐ may pick one or more). See definitions below:** • Participation: The intervention promotes or formalises continuous citizen input in the design and implementation of public services, processes or policies. • Inclusion: The intervention promotes access to services for the whole community, particularly that of marginalised and vulnerable groups such as women, ethnic minorities or lesbian, gay bisexual, transgender and intersex (LGBTI) people. • Transparency: The intervention involves the disclosure and/or dissemination of information (rules, plans, processes, prices and actions) regarding public services or institutions. • Accountability: The intervention encompasses monitoring to encourage or actively hold individuals, public service providers and institutions responsible for executing their powers and mandates according to a certain standard.	1 = Participation 2 = Inclusion 3 = Transparency 4 = Accountability
	**Sector**	**Indicate the sector covered by the intervention (multiple choice ‐ may pick one or more)**	1 = Health 2 = Public financial management 3 = Natural resource management 4 = Disaster response / management 5 = Social protection 6 = Justice 7 = Environment / climate change
	**Intervention description**	**Provide descriptive details about the intervention**	**Open answer**
	**Objectives of intervention**	**State any objectives stated in study or other document**	**Open answer**
	**Intervention scale**	With what level of government is the intervention working? N.B interventions to shape PITA at the national level are very different from those striving to shape PITA at sub‐national levels in terms of level of effort and range of stakeholders and power dynamics	1 = National 2 = Regional government 3 = Local level government
	**Intervention development**	**To what extent is the intervention locally‐ developed / demanded or donor created? Please present any information presented in the paper(s), N/A if no information presented**	**Open answer**
	**Intervention implementing agency**	**Who is implementing the intervention? State the name (and department) of the implementing agency.**	**Open answer**
	**Intervention funding agency**	**Type of funder**	1=Government 2=NGO 3=Multilateral/bilateral organisation 4= Foundation 5= Private sector 6= Other
	**Intervention funding agency**	**Name of intervention funding agency**	**Open answer**
	**Intervention target group**	**What were the characteristics of beneficiaries used to target the intervention?**	**Open answer**
	**Targeting methods**	**How were beneficiaries targeted for the programme (Eg: how was the targeting implemented)?**	**Open answer**
	**Intervention start**	**Start date (if not stated, state study date) of intervention**	**XX/XXXX**
	**Intervention end**	**State end date (if ongoing state ongoing)**	**XX/XXXX**
	**Intervention length**	**Start intervention length (months)**	**State number of months**
**Equity**	**Consideration of equity**	**Does the study consider equity?**	**1=Yes 2=No**
	**Equity methods**	**How does the study consider equity?**	1=intervention target a disadvantaged group 2=study measures inequality 3=sub‐group analysis by dimension of inequity
	**Equity dimension**	**What dimension(s) of equity does the study consider? PROGRESS + indicators (multiple choice ‐ may pick more than one)**	1 = place of residence 2 = race / ethnicity 3 = occupation 4 = gender 5 = religion 6 =education 7 = socioeconomic status 8 = social capital 9 = age 10 = disability 11 = sexual orientation
**Process and implementation**	**Information about program take‐up**	Is there any information about program take‐up? Commentary by authors should be used when information on program take / up etc. is not backed up by some sort of research / when the authors do not report that/how they collected data to assess these areas.	**1=Yes, commentary from author; 2= Yes, formally assessed, 3=No;**
	**Methods of assessing take‐up**	**Which methods are used to assess program take‐up?**	1= Observation by intervention staff 2= Reporting by participants 3= Other 4= Commentary from author 9= Not measured
	**Results of the assessment of take‐up**	**What is the result/ information provided of the assessment of program take‐up?**	**Open answer**
	**Information about program adherence (among beneficiaries)**	Is there any information about program adherence (among beneficiaries)? Commentary by authors should be used when information on program adherence etc. is not backed up by some sort of research / when the authors do not report that/how they collected data to assess these areas.	**1=Yes, commentary from author; 2= Yes, formally assessed, 3=No;**
	**Methods of assessing adherence**	**Which methods are used to assess program adherence?**	1= Observation by intervention staff 2= Reporting by participants 3= Other 4= Commentary from author 9= Not measured
	**Results of the assessment of adherence**	**What is the result/ information provided of the assessment of program adherence?**	**Open answer**
	**Information about implementation fidelity / intervention delivery quality**	Is there any information on implementation fidelity/ intervention delivery quality? Commentary by authors should be used when information on program adherence etc. is not backed up by some sort of research / when the authors do not report that/how they collected data to assess these areas.	**1=Yes, commentary from author; 2= Yes, formally assessed, 3=No;**
	**Methods of assessing intervention fidelity**	**Which methods are used to assess implementation fidelity/ intervention delivery quality**	**1= Observation by intervention staff2= Reporting by participants 3= Other4= Commentary from author9= Not measured**
	**Results of the assessment of intervention fidelity**	**What is the result/ information provided of the assessment of implementation fidelity/ intervention delivery quality**	**Open answer**
	**Other description of process / implementation factors**	**Any other description of process / implementation factors not covered above**	**Open answer**
**Contextual barriers / enablers**	**Causal mechanisms / barriers and enablers**	**Does the study identify any causal mechanisms / barriers and enablers related to context (not included above)?**	**1=Yes, commentary from author; 2= Yes, formally assessed, 3=No;**
	**Methods**	**How are these identified?**	1= Observation by intervention staff 2= Reporting by participants 3= Other 4= Commentary from author
	**Results**	**Report here any material relevant to causal mechanisms and barriers and enablers**	**Open answer**
**Cost**	**Cost**	**Are any unit cost data / cost‐effectiveness estimates provided?**	**1=Yes 2=No**
	**Cost details**	**If yes, report any details of unit cost and/or total cost. Please also report year and currency.**	**Open answer**
**External Validity**	**Length of study**	**Length of study in months (Where study length not reported, code as length of intervention, noting that in brackets)**	**# months, if not reported N/A**
	**Efficacy or effectiveness trial**	**Was the intervention implemented under “real world” conditions? By real world we mean a programme implemented independently of the evaluation, either by government, NGO or international agency**	**1=Yes 2=No 9= N/A**
	**Personell implementing the programme**	**Who was in charge of implementing the program?**	**1=PI/ researchers (study authors); 2= implementing agency staff, 3= external agency (eg: survey firm); 4=Others; 8= Not clear**
	**Sampling frame for the study**	**State the sampling frame (list of all those within a population who can be sampled, ie. households, communities) for selection of study participants (i.e. Census, etc).**	**Open answer**
	**Author discussion of external validity**	**Do the authors discuss or explicitly address generalisability / applicability?**	**Open answer**
	**Program theory**	**Do the authors make explicit reference to program theory, theory of change or similar?**	**1=Yes 2=No 8=Not clear**
	**Program theory**	**Report any description/statement of program theory as stated by author(s).**	**Open answer**
	**Program theory**	**Is the study using theory to inform the evaluation design and analysis?**	**Open answer ‐ describe if and how the authors use theory in the evaluation. Do they for example use it to inform data collection? Do they do any causal chain analysis?**

**Effect size data coding**

**Table:**

	**Description**	**Question**	**Coding**
**ID**	**Unique study identification #**		**For example, PITA001**
	**First author ‐ impact evaluation**	**Surname**	**Open answer**
**Outcome for effect size (answer for all studies)**	**Outcome**	**Which outcome is being coded?**	1 = Knowledge and attitudes about services 2 = Access to services 3 = Use of services 4 = Performance or quality of services 5 = Income or poverty status 6 = Health outcome 7 = Nutritional status / food security 8 = Resilience (including coping strategies) 9 = Environmental (non‐human) outcome 10 = Social and psychological outcomes 11 = Other
	**Definition of outcome**	**Please provide the authors definition of the outcome (including description of the sub‐group if relevant)**	**Open answer**
	**Follow up period**	**What is the follow up period of this outcome after initiation of intervention? (specify in months)**	**Number of months**
	**Sub‐group analysis**	**Is this effect size data for a sub‐group?**	1 = No 2 = Yes
	**Sub‐group analysis description**	**If yes to question 2, which type of sub‐group?**	**Open answer ‐ this can include separate samples for gender, income, place of residence**
	**Effect size location**	**Which page(s) contain the effect size data?**	**Open answer**
	**Data to be extracted**	**Which type of data to be extracted?**	1 = Continuous ‐ means and SDs 2 = Continuous ‐ mean difference and SD 2 = Dichotomous outcome ‐ proportions 3 = Regression data
**Effect size data (answer for all studies)**	**Sample size metric**	**Sample size unit of analysis**	1= Individual 2= Household 3= Group (e.g. community organisation) 4= Village 5 = Other 6 = Not clear
	**Treatment effect estimated**	**What treatment effect is estimated?**	1=ITT 2=ATET 3=ATE 4=LATE
	**Sample size (treatment)**	**Initial sample size treatment group**	**#**
	**Sample size (control)**	**Initial sample size control group**	**#**
	**Sample size (total)**	**Initial sample size total**	**#**
	**Observations (treatment)**	**Number of treatment observations after attrition / follow up**	**#**
	**Observations (control)**	**Number of control observations after attrition / follow up**	**#**
	**Observations (total)**	**Total number of control observations after attrition / follow up**	**#**
**Outcome data ‐ if continuous (Means and SDs)**	**Baseline outcome treatment**	**State result of baseline outcome for treatment group**	**#**
	**SD Baseline outcome treatment**	**State SD of baseline outcome measure for treatment group**	**#**
	**Baseline outcome control**	**State result of baseline outcome for control group**	**#**
	**SD Baseline outcome control**	**State SD of baseline outcome measure for control group**	**#**
	**Outcome in treatment post intervention**	**State result of post intervention outcome for treatment group**	**#**
	**SD Outcome in treatment post intervention**	**State SD of post intervention outcome measure for treatment group**	**#**
	**Outcome in control post intervention**	**State result of post intervention outcome for control group**	**#**
	**SD Outcome in control post intervention**	**State SD of post intervention outcome measure for control group**	**#**
	**Outcome in treatment 1st follow up**	**State result of 1st follow up outcome measure for treatment group**	**#**
	**SD Outcome in treatment 1st follow up**	**State SD 1st follow up outcome measure for treatment group**	**#**
	**Outcome in control 1st follow up**	**State result of 1st follow up outcome measure for treatment group**	**#**
	**SD Outcome in control 1st follow up**	**State SD 1st follow up outcome measure for treatment group**	**#**
**Outcome data ‐ If continuous (Mean difference and SD / SE at follow up)**	**Mean difference at follow up**	**State mean difference**	**#**
	**SD at follow up**	**State SD at follow up**	**#**
	**SE**	**State SE**	**#**
**Outcomes data ‐ if dichotomous (Proportions r)**	**Baseline number with outcome in treatment**	**State result of baseline outcome for treatment group**	**#**
	**Proportion with outcome at baseline in treatment**	**State proportion with outcome at baseline in treatment**	**#**
	**Baseline number with outcome in control**	**State result of baseline outcome for treatment group**	**#**
	**Proportion with outcome at baseline in control**	**State proportion with outcome at baseline in control**	**#**
	**Number with outcome in treatment post intervention**	**State number with outcome post intervention for treatment group**	**#**
	**Proportion with outcome in treatment group post intervention**	**State proportion with outcome post intervention in control group**	**#**
	**Number with outcome in control post intervention**	**State number with outcome post intervention for control group**	**#**
	**Proportion with outcome in control group post intervention**	**State proportion with outcome post intervention in control group**	**#**
	**Number with outcome in treatment 1st follow up**	**State number with outcome at 1st follow up for treatment group**	**#**
	**Proportion with outcome in treatment group 1st follow up**	**State proportion with outcome at 1st follow up in treatment group**	**#**
	**Number with outcome in control 1st follow up**	**State number with outcome at 1st follow up for control group**	**#**
	**Proportion with outcome in control group 1st follow up**	**State proportion with outcome at 1st follow up in control group**	**#**
**Regression data**	**OLS**	**OLS used?**	**1=Yes 2=No**
	**Logistic**	**Logistic used?**	**1=Yes 2=No**
	**Type of logistic**	**What type of logistic regression?**	**1=binomial 2=multinomial**
	**GLS/WLS**	**GLS or WLS used?**	**1=Yes 2=No**
	**other regression types**	**Other regression type used? Specify**	**open answer**
	**continuous outcome**	**Is the outcome continuous?**	**1=Yes 2=No**
	**dichotomous outcome**	**Is the outcome dichotomous?**	**1=Yes 2=No**
	**multiple outcome categories**	**Does the outcome have more than 2 categories?**	**1=Yes 2=No 3=Continuous**
	**type of coefficient**	**What is the coefficient type?**	**1=raw 2=standardized 3=other**
	**coefficient**	**What is the coefficient estimate?**	**#**
	**pooled standard deviation of outcome**	**What is the pooled standard deviation of the outcome?**	**#**
	**standard error**	**What is the standard error of the coefficient estimate?**	**#**
	**t test**	**What is the t statistic associated with the focal predictor?**	**#**

## Draft critical appraisal tool

*Note: this is a draft coding risk of bias tool and may be adapted following further piloting*.

**Study design classifier and risk of bias tool for quantitative impact evaluations (studies included to answer questions 1, 2 and 3)**

**Table:**

** **	**Description**	**Question**	**Coding**
**ID**	**Unique study identification #**	**Study**	**For example, PITA001**
	**Paper**	**Surname / year of first author of paper for effect size data extraction**	**Open answer**
**Research methods ‐ study design and risk of bias**	**Design type**	**What type of study design is used?**	1= Randomised controlled trial (RCT) (random assignment to households/individuals) or quasi‐RCT 2= Cluster‐RCT (quasi‐RCT) 3= Natural experiment: randomised or as‐if randomised 4= Natural experiment: regression discontinuity (RD) 5 = CBA (non‐randomised assignment with treatment and contemporaneous comparison group, baseline and endline data collection) – individual repeated measurement 6= CBA pseudo panel (repeated measurement for groups but different individuals) 7= Interrupted time series (with or without contemporaneous control group) 8= Panel data, but no baseline (pre‐test) 9 = Comparison group with endline data only
	**Methods used for analysis**	**Which methods are used to control for selection bias and confounding?**	1= Statistical matching (PSM, CEM, covariate matching) 2= Difference in differences (DID) estimation methods 3= IV‐regression (2‐stage least squares or bivariate probit) 4=Heckman selection model 5= Fixed effects regression 6= Covariate adjusted estimation 7= Propensity weighted regression 8= Comparison of means 9 = Other
	**Design and analysis method description**	**Briefly describe the study design and analysis method undertaken by the authors**	**Open answer**
	**Unit of analysis**	**Is unit of analysis in cluster allocation addressed in standard error calculation (RCT and NRS)?**	**1=Yes 2=No 3=Not reported/unclear 4=Not applicable**
	**Method used to address differences between UoA and unit of data collection**	**Briefly describe methods used to adjust standard errors to account for correlation of observations within clusters (e.g. cluster‐robust standard errors reported)**	**Open answer**
	**Type of comparison group**	**Indicate type of comparison group**	1=No intervention (service delivery as usual) 2=Other PITA intervention 3=Pipeline (wait‐list) control (still service delivery as usual)
	**Assignment mechanism**	**1: Mechanism of assignment: was the allocation or identification mechanism random or as good as random?**	**1= Yes, 2 = Probably Yes, 3 = Probably No, 4 = No, 8 = Unclear**
	**Assignment justification**	Justification for coding decision (Include a brief summary of justification for rating, mentioning your response to all sub questions, cite relevant pages)	**Open answer**
	**Confounding**	**Group equivalence: was the method of analysis executed adequately to ensure comparability of groups throughout the study and prevent confounding**	**1= Yes, 2 = Probably Yes, 3 = Probably No, 4 = No, 8 = Unclear**
	**Confounding justification**	Justification for coding decision (Include a brief summary of justification for rating, mentioning your response to all sub questions, cite relevant pages)	**Open answer**
	**Selection bias**	**Was any differential selection into or out of the study (attrition bias) adequately resolved?**	**1= Yes, 2 = Probably Yes, 3 = Probably No, 4 = No, 8 = Unclear**
	**Selection bias justification**	Justification for coding decision (Include a brief summary of justification for rating, mentioning your response to all sub questions, cite relevant pages)	**Open answer**
	**Spill‐overs, cross‐overs and contamination**	**2: Spill‐overs, cross‐overs and contamination: was the study adequately protected against spill‐overs, cross‐overs and contamination?**	**1= Yes, 2 = Probably Yes, 3 = Probably No, 4 = No, 8 = Unclear**
	**Spill‐overs justification**	Justification for coding decision (Include a brief summary of justification for rating, mentioning your response to all sub questions, cite relevant pages)	**Open answer**
	**Motivation bias**	**Was the process of being observed free from motivation bias (e.g. Hawthorne effects)?**	**1= Yes, 2 = Probably Yes, 3 = Probably No, 4 = No, 8 = Unclear**
	**Motivation justification**	Justification for coding decision (Include a brief summary of justification for rating, mentioning your response to all sub questions, cite relevant pages)	**Open answer**
	**Outcome reporting**	**3: Outcome reporting: was the study free from selective outcome reporting?**	**1= Yes, 2 = Probably Yes, 3 = Probably No, 4 = No, 8 = Unclear**
	**Outcome reporting**	Justification for coding decision (Include a brief summary of justification for rating, mentioning your response to all sub questions, cite relevant pages)	**Open answer**
	**Analysis reporting**	**4: Analysis reporting: was the study free from selective analysis reporting?**	**1= Yes, 2 = Probably Yes, 3 = Probably No, 4 = No, 8 = Unclear**
	**Analysis reporting**	Justification for coding decision (Include a brief summary of justification for rating, mentioning your response to all sub questions, cite relevant pages)	**Open answer**
	**Performance bias**	**5: Performance bias: was the process of being observed free from motivation bias?**	**1= Yes, 2 = Probably Yes, 3 = Probably No, 4 = No, 8 = Unclear**
	**Performance bias**	Justification for coding decision (Include a brief summary of justification for rating, mentioning your response to all sub questions, cite relevant pages)	**Open answer**
	**Other bias**	**6: Other risks of bias: Is the study free from other sources of bias? Including around measurement of the intervention**	**1= Yes, 2 = Probably Yes, 3 = Probably No, 4 = No, 8 = Unclear**
	**Other bias**	Justification for coding decision (Include a brief summary of justification for rating, mentioning your response to all sub questions, cite relevant pages)	**Open answer**
	**Blinded participants**	**Blinding of participants?**	**1=Yes 2=No 9= N/A**
	**Blinded observers**	**Blinding of outcome assessors?**	**1=Yes 2=No 9= N/A**
	**Blinded analysts**	**Blinding of data analysts?**	**1=Yes 2=No 9= N/A**
	**Method used to blind**	**Describe method(s) used to blind**	**Open answer (including describe method of placebo control)**
